# A functional map of HIV-host interactions in primary human T cells

**DOI:** 10.1038/s41467-022-29346-w

**Published:** 2022-04-01

**Authors:** Joseph Hiatt, Judd F. Hultquist, Michael J. McGregor, Mehdi Bouhaddou, Ryan T. Leenay, Lacy M. Simons, Janet M. Young, Paige Haas, Theodore L. Roth, Victoria Tobin, Jason A. Wojcechowskyj, Jonathan M. Woo, Ujjwal Rathore, Devin A. Cavero, Eric Shifrut, Thong T. Nguyen, Kelsey M. Haas, Harmit S. Malik, Jennifer A. Doudna, Andrew P. May, Alexander Marson, Nevan J. Krogan

**Affiliations:** 1 Department of Microbiology and Immunology, University of California, San Francisco, CA 94143 USA; 2 Diabetes Center, University of California, San Francisco, CA 94143 USA; 3 Gladstone Institutes, San Francisco, CA 94158 USA; 4 Innovative Genomics Institute, University of California, Berkeley, CA 94720 USA; 5 Division of Infectious Diseases, Northwestern University Feinberg School of Medicine, Chicago, IL 60611 USA; 6 Center for Pathogen Genomics and Microbial Evolution, Institute for Global Health, Northwestern University Feinberg School of Medicine, Chicago, IL 60611 USA; 7 Department of Cellular and Molecular Pharmacology, University of California, San Francisco, CA 94158 USA; 8 Quantitative Biosciences Institute, QBI, University of California, San Francisco, CA 94158 USA; 9 Chan Zuckerberg BioHub, San Francisco, CA 94158 USA; 10 Howard Hughes Medical Institute, Fred Hutchinson Cancer Research Center, Seattle, WA 98109 USA; 11 Howard Hughes Medical Institute, University of California, Berkeley, CA 94720 USA; 12 Department of Molecular and Cell Biology, University of California, Berkeley, CA 94720 USA; 13 MBIB Division, Lawrence Berkeley National Laboratory, Berkeley, CA 94720 USA; 14 Department of Chemistry, University of California, Berkeley, CA 94720 USA; 15 Department of Medicine, University of California, San Francisco, CA 94143 USA; 16 UCSF Helen Diller Family Comprehensive Cancer Center, University of California, San Francisco, CA 94158 USA

**Keywords:** Virus-host interactions, Systems virology

## Abstract

Human Immunodeficiency Virus (HIV) relies on host molecular machinery for replication. Systematic attempts to genetically or biochemically define these host factors have yielded hundreds of candidates, but few have been functionally validated in primary cells. Here, we target 426 genes previously implicated in the HIV lifecycle through protein interaction studies for CRISPR-Cas9-mediated knock-out in primary human CD4+ T cells in order to systematically assess their functional roles in HIV replication. We achieve efficient knockout (>50% of alleles) in 364 of the targeted genes and identify 86 candidate host factors that alter HIV infection. 47 of these factors validate by multiplex gene editing in independent donors, including 23 factors with restrictive activity. Both gene editing efficiencies and HIV-1 phenotypes are highly concordant among independent donors. Importantly, over half of these factors have not been previously described to play a functional role in HIV replication, providing numerous novel avenues for understanding HIV biology. These data further suggest that host-pathogen protein-protein interaction datasets offer an enriched source of candidates for functional host factor discovery and provide an improved understanding of the mechanics of HIV replication in primary T cells.

## Abstract (web summary)

Here, Hiatt et al. report the knock-out of over 400 genes in primary CD4+ T cells to assess their functional role in HIV replication, finding 86 initial candidates of which 47 are validated as HIV host factors, including 23 with restrictive activity.

## Introduction

Improved understanding of the molecular mechanisms underlying HIV replication, persistence, and pathogenesis is critical for the development of new therapeutic and curative strategies^1–4^. All viruses rely on and manipulate the molecular architecture of their host’s cells for successful replication and dissemination. Host proteins and complexes that are necessary for or that facilitate viral replication are known as dependency factors, while those that inhibit viral replication are known as restriction factors^5–9^. Inhibiting the action of dependency factors or enhancing the activity of restriction factors may significantly curtail viral replication and spread, and thus, these factors may serve as targets for therapeutic intervention. For example, the antiretroviral drug Maraviroc, used for the treatment of HIV infection, acts through antagonism of the dependency factor CCR5, which is required for viral entry^10^. Likewise, novel drugs that disrupt the integrity of incoming HIV core particles and their interactions with host factors are under clinical development^11,12^.

While targeted mechanistic studies have identified several well-characterized host factors influencing HIV replication, systematic attempts to identify and catalog host factors through genetic means have yielded variable results with limited consensus. For example, four genome-wide RNA-interference (RNAi) screens for HIV host factors have been previously published, each identifying roughly 250–400 genes impacting replication for a hit rate that ranges from 0.46 to 1.83% of screened targets (Table 1)^13–17^. Despite these herculean efforts, not a single gene was found in common between all four datasets and no more than three genes were found in common between any three datasets. These broad differences have been attributed, at least in part, to inherent limitations in the RNAi-based approaches used, as well as to the differences in the immortalized cell-line models employed^14,18^.

**Table 1:** Comparison of results in this study to previously published screens^13,15–17,19,20^.

Study	Hits	Genes targeted	Hit rate	Gene list description	Technology	Pooled or arrayed	Cell type
Yeung et al.	252	54509	**0.46%**	54,509 human transcripts, including ESTs	shRNA	Pooled	Jurkat
Brass et al.	386	21,121	**1.83%**	21,121 pools of 4 siRNAs per gene (genome wide)	siRNA	Arrayed	TZM-bl
Konig et al.	294	20000	**1.47%**	arrayed genome-wide siRNA library targeting ∼20,000 human genes	siRNA	Arrayed	293 T
Zhou et al.	311	19709	**1.58%**	siRNA library targeting 19,709 genes with pools of 3 siRNAs per gene	siRNA	Arrayed	HeLa P4/R5
OhAinle et al.	15	1905	**0.79%**	Interferon-stimulated genes in cell types relevant to HIV infection	CRISPR	Pooled	THP-1
Park et al.	5	18543	**0.03%**	Genome-wide protein-coding genes	CRISPR	Pooled	GXRCas9 T cell line
Hiatt et al.	47	426	**11.03%**	HIV interactome (Jager et al.)	CRISPR RNPs	Arrayed	Primary human CD4+ T cells

Bold values reflect the number of hits over the number of genes targeted as indicated.

More recently, two CRISPR–Cas9-based screens for HIV-dependency factors^19^ and restriction factors^20^ have been reported, both of which also relied on immortalized cell-line models (Table 1). Unlike the RNAi studies, which formatted their screens in large arrays of independent wells, these CRISPR-based studies leveraged a pooled approach whereby iterative rounds of selection were used to identify hits that conferred the strongest phenotype or selective advantage in the study. While effective as discovery-based platforms, such pooled approaches often rely on indirect phenotypic readouts that may favor the most impactful perturbations, limiting sensitivity to detect mild phenotypes^21^. Furthermore, none of the reports above included a systematic validation of the genetic perturbations, which subsequently limited results to only those genes that gave a positive phenotype. In other words, the difference between a technical inability to knock down or knock out a gene target and a true biological finding of no impact on infection could not be resolved. A genome-wide, arrayed CRISPR screen for HIV host factors has not yet been reported, likely reflecting the cost and complexity of such a project.

Most bona fide HIV host factors that have been described mechanistically to date directly interact with a viral protein, nucleic acid, or ribonucleoprotein complex. As such, a number of studies have leveraged biochemical approaches to characterize HIV virion-associated proteins and virus–host protein–protein interactions (PPIs)^22–25^. In a previous study, we employed an affinity-purification coupled with mass spectrometry (AP-MS) approach to identify 435 HIV–human PPIs in two human cell lines^23^. Several well-documented interactions were validated, including those between Tat and the dependency factors CDK9 and CCNT1^26,27^, and those between Vif and the dependency factors ELOB and ELOC^28–30^. Since then, a number of novel interactors have been genetically and biochemically validated as host factors, including CBFβ^31^, PJA2^32^, UBE2O^33^, and AMBRA1^34^. Nevertheless, a vast majority of these interactions have yet to be characterized functionally or mechanistically interrogated.

Over the past several years, we have developed and optimized a high-throughput system for CRISPR–Cas9 gene editing in primary CD4+ T cells^35–37^. In vitro-assembled CRISPR–Cas9 ribonucleoprotein complexes (RNPs) are electroporated into primary T cells in arrayed format allowing for the reproducible and efficient knockout of gene targets. Edited cells retain high viability and susceptibility to infection, allowing for the generation of quantitative, arrayed data on the impact of host-factor knockout on HIV replication^36,37^. Coupled with deep sequencing to validate knockout efficiency, this technology has the potential to overcome many of the previous limitations to the systematic identification of HIV host factors, but thus far, its use has been limited to targeted interrogation of only a handful of genes (i.e., CYPA/TRIM5α^38^ and ARIH2^39^).

In this report, we use CRISPR–Cas9 RNPs for systematic targeting of 426 previously identified PPIs in primary CD4+ T cells to determine their functional impact on HIV replication^23^. We performed deep sequencing to quantify allelic knockout efficiency for each perturbation (see also^40^), and monitored viral infection over seven days following HIV-1 challenge. Linked genotypic and phenotypic data allowed for discrimination between genes that were not effectively perturbed and those that had no impact on infection. In total, 86 candidate host factors were identified, nearly half of which recapitulated known biology. Of these 86 candidate genes, 47 host factors were validated by gene editing in independent donors using multiplexed RNPs, including 23 factors with restrictive activity. Notably, this proteomics-to-genetics approach resulted in a greater than 10% hit rate, demonstrating that PPI datasets represent an enriched fraction of host factors (Table 1). This strategy may be effective at focusing high-quality, arrayed screening experiments for host-factor identification in primary cell types in the future. Continued exploration of protein interactions and functional mechanisms in primary human cells will be vital to resolve outstanding questions in the field and derive consensus on the host factors that may be leveraged for future therapeutics.

## Results

### Arrayed knockout of HIV–human PPIs

**Fig. 1:**
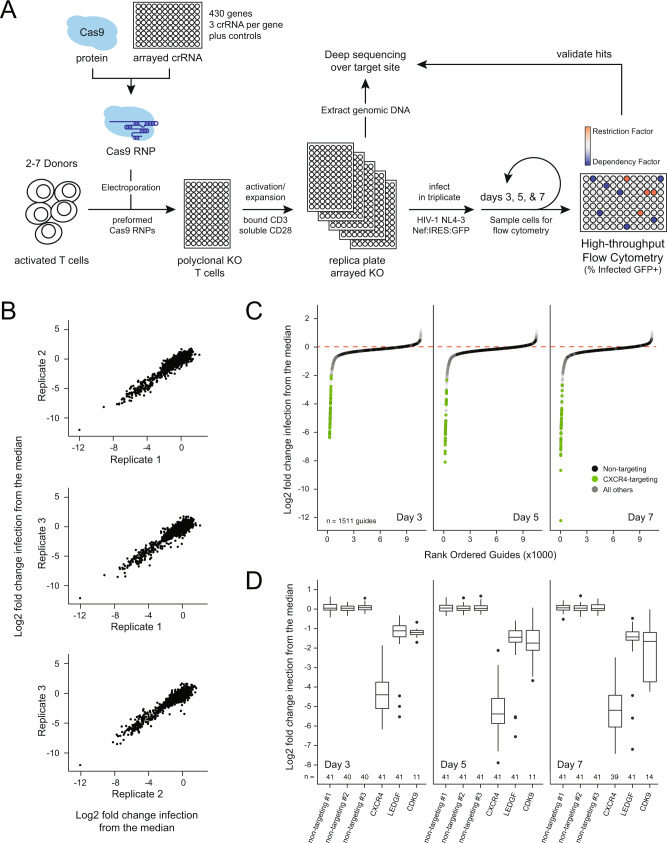
An arrayed screening pipeline for HIV host-factor identification. **A** Schematic of the HIV host-factor screen design using high-throughput CRISPR–Cas9 gene editing in primary CD4+ T cells. **B** Scatterplots of the log_2_ fold change in infection relative to the plate median for each gRNA after data processing compared across technical replicates. **C** S-curve plots of the log_2_ fold change in infection relative to the plate median for each gRNA in every donor, rank-ordered by timepoint. The dashed red line indicates median; green dots represent *CXCR4*-targeting control gRNA and black dots represent nontargeting gRNA. **D** Box-and-whisker plot of the distribution of log_2_-normalized HIV infection rates for each control gRNA at each timepoint. Total *n* after filtering is indicated below each box; center line, median; box limits, upper and lower quartiles; whiskers, 1.5x interquartile range; points, outliers.

To define the functional contribution of previously identified HIV–human PPIs to HIV replication^23^, we aimed to employ a CRISPR-Cas9 RNP approach to knock out 435 genes in primary CD4+ T cells from multiple, independent human-blood donors (Fig. 1A). Three independent guide RNAs (gRNAs) per gene were arrayed across a total of eighteen 96-well plates, targeting 426 of the 435 genes from the protein–protein-interaction dataset, plus two additional genes encoding previously described host factors CD4 and HEXIM1^41,42^ (Supplementary Data 1). Nine genes could not be targeted for specific deletion due to extensive sequence homology with related family members; two members of the HIST1H3 gene family (HIST1H3D and HIST1H3I) were selected for knockout from the larger gene family (Supplementary Data 1). Each 96-well plate included three distinct, nontargeting, negative-control gRNAs that do not align to any PAM-adjacent region of the human genome and should not cause any Cas9-induced double-strand breaks. We also included three previously validated, positive-control gRNAs on each plate targeting known HIV host factors: the HIV coreceptor *CXCR4*, which is required for viral entry of CXCR4-tropic viruses like the NL4–3 strain used here^43–45^; *LEDGF*, which facilitates viral integration^46,47^; and the transcription factor *CDK9*, which is hijacked by the accessory protein Tat to promote HIV transcription^26,27^.

Cas9 RNPs were generated as previously described and frozen in 96-well arrays^36,37,48^. The arrayed RNPs were electroporated into 400,000 activated CD4+ T cells per well. Each electroporation was repeated in cells from at least two distinct biological donors (total of 18 donors used over the entire experiment, median of 147 unique gRNAs tested per donor, see also Supplementary Fig. 1a). After allowing six days for DNA repair, protein depletion, and cell recovery, genomic DNA and protein samples were harvested from each culture for determination of knockout efficiency. The following day, cells were challenged in triplicate with replication-competent HIV-1 NL4–3 Nef:IRES:GFP^49^. Infection rate (percent GFP+ cells) and cell count were monitored by flow cytometry at days 3, 5, and 7 post infection to capture HIV host factors that act both early and late in the replication cycle (Fig. 1A, Supplementary Fig. 2a). To facilitate comparison of infection rate across different plates and donor samples, the data were filtered, corrected for edge effects, and normalized to the median infection percentage of each plate to calculate a log_2_ fold change in percent HIV infection (Supplementary Fig. 2b). The resultant fold changes were strongly correlated across technical triplicates and used to calculate mean and standard deviation for subsequent analyses (Fig. 1B). Samples with low cell counts or high variability in either cell count or infection rate were removed from further analysis (154 of 31,209 samples, Supplementary Fig. 2b, c).

Each gRNA was ranked based on the log_2_ fold change in HIV-infection rate relative to the plate median (Fig. 1C). The majority of gRNAs clustered closely around the plate median and the nontargeting controls (black dots, Fig. 1C), indicating that either the gRNA was ineffective at knocking out the targeted gene, or that the targeted gene does not influence HIV replication in activated CD4+ T cells. The six control gRNAs resulted in highly reproducible changes in infection rate across all donors and plates, with each nontargeting control clustering tightly at the plate median (Fig. 1D). Knockout of CXCR4 (green dots, Fig. 1C), resulted in strong decreases in infection rates at all three timepoints, as did knock out of LEDGF and CDK9 (Fig. 1D). Notably, CDK9, a component of the positive transcription-elongation factor b (P-TEFb) complex, is critical for both viral and cellular transcription^26,27,50^; knockout of this factor yielded diminished cell viability with several wells excluded from analysis due to viability filtering (Fig. 1D). Overall, more gene knockouts resulted in decreased rather than increased infection (Fig. 1C). This likely reflects the relative rarity of restriction factors compared with dependency factors^5^, the greater potential for nonspecific disruption of T-cell architecture/activation, as well as the capacity of wild-type HIV-1 to evade host defenses in CD4+ T cells. In other words, knockout of virally countered restriction factors would not be expected to have an observable phenotype on the replication of wild-type viruses. For example, the antiviral restriction factor APOBEC3G is already counteracted by the HIV Vif protein^30,51^, so knockout of APOBEC3G would not be expected to influence the replication of a wild-type virus.

### Quantification of mutational efficiency

**Fig. 2:**
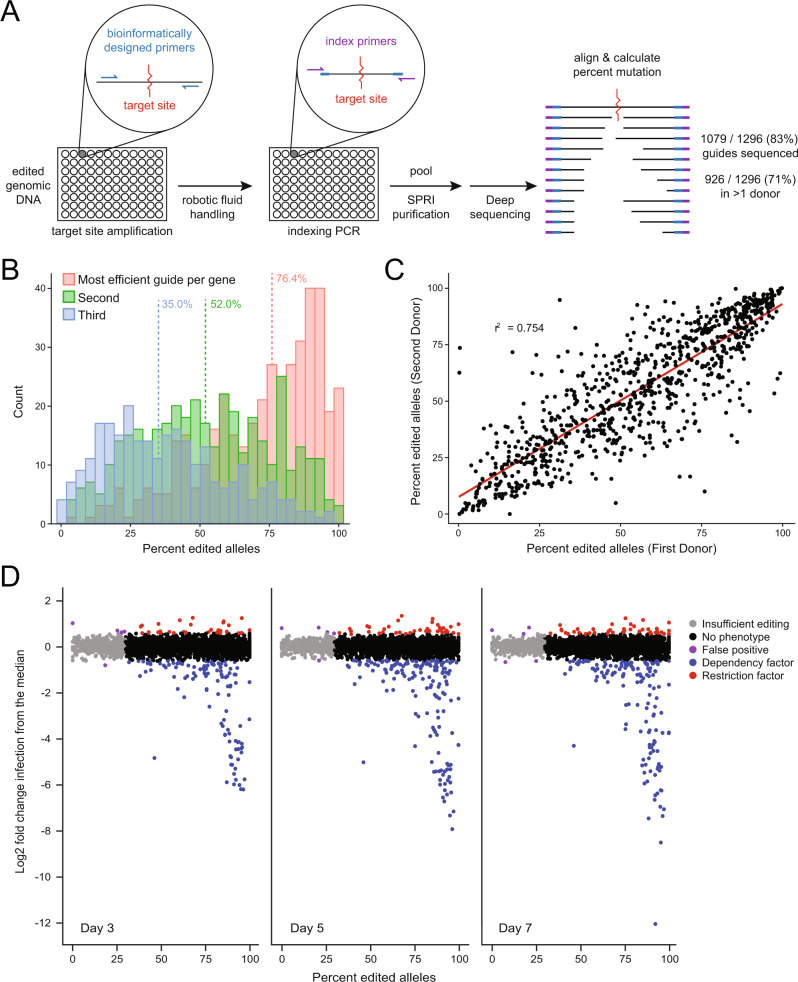
Next-generation sequencing reveals efficient and reproducible editing. **A** Schematic of the deep-sequencing approach to quantify mutational efficiency for each gRNA. **B** Histograms depicting the allelic knockout efficiency for the most efficient (red), middle (green), and least-efficient (blue) gRNA per gene. Median values of each group are indicated by dashed vertical lines. **C** Representative scatterplot of mutational efficiency in a subset of randomly selected gRNA across two independent donors (*r*^2^ mean = 0.745, range 0.722–0.772 over 100 randomizations). **D** Scatterplot of mutational efficiency versus the log_2_ fold change in infection relative to the plate median for each gRNA at days 3 (left), 5 (middle), and 7 (right). Each gRNA dot is colored by phenotypic outcome based on empirically determined cutoffs to achieve a 1% false-positive rate.

To measure the mutational efficiency of each gRNA, we next quantified the fraction of alleles knocked out in each reaction in each donor using high-throughput, next-generation amplicon sequencing (Fig. 2A). Repair of the CRISPR–Cas9-induced double-strand breaks by the endogenous DNA-repair machinery resulted in variable, but nonrandom sequences at the cut site in each polyclonal pool of cells^40^. Alignment of these reads allowed us to calculate percent mutational efficiency, defined as the fraction of aligned reads that resulted in a frameshift mutation or an insertion or deletion of more than two amino acids. Using this method, we were able to calculate the mutational efficiencies for 83% (1079 out of 1296) of the gRNA used in the study in at least one blood donor.

The most efficient guide for each gene had a median allelic mutational efficiency of 76.4%, with several guides editing all observed alleles (Fig. 2B). Including controls, of the 430 genes targeted, 364 (85%) had sequence-confirmed disruption of at least 50% of alleles with at least one gRNA (Supplementary Fig. 1b). The editing efficiency of each gRNA was highly correlated between donors (pairwise *r*^2^ range 0.67–0.99, mean = 0.88, Fig. 2C, Supplementary Fig. 1c). In silico off-target analysis found that over 88% of gRNA used in the study had minimal predicted off-target effects, and verified that every gene is represented by at least one gRNA with low off-target probability^52^ (Supplementary Data 1).

### Identification of candidate host factors

Plotting mutational efficiency versus the relative HIV-infection rate revealed editing-dependent changes at each timepoint (Fig. 2D). Overall, gRNAs with poor editing efficiency did not impact HIV infectivity, while a subset of gRNAs with high efficiency yielded marked effects. To determine the minimum percent editing required to detect a change in HIV infectivity, CXCR4-knockout cells were mixed with nontargeting control cells at fixed ratios from 0 to 100% and challenged with HIV-1 as above. At each timepoint, significant decreases in infection were observed when at least 30% of the population consisted of edited cells (Supplementary Fig. 3a). The same held true when titrating LEDGF- or CDK9-knockout cells (Supplementary Fig. 3b, c). While this experiment quantifies knockout on a per-cell rather than per-allele basis, these data suggest that a minimum of 30% allelic editing is required to cause an observable phenotype in this assay when targeting factors are absolutely required for viral replication. Given these results, we considered any phenotypic variation below this editing-efficiency threshold to be noise.

**Fig. 3:**
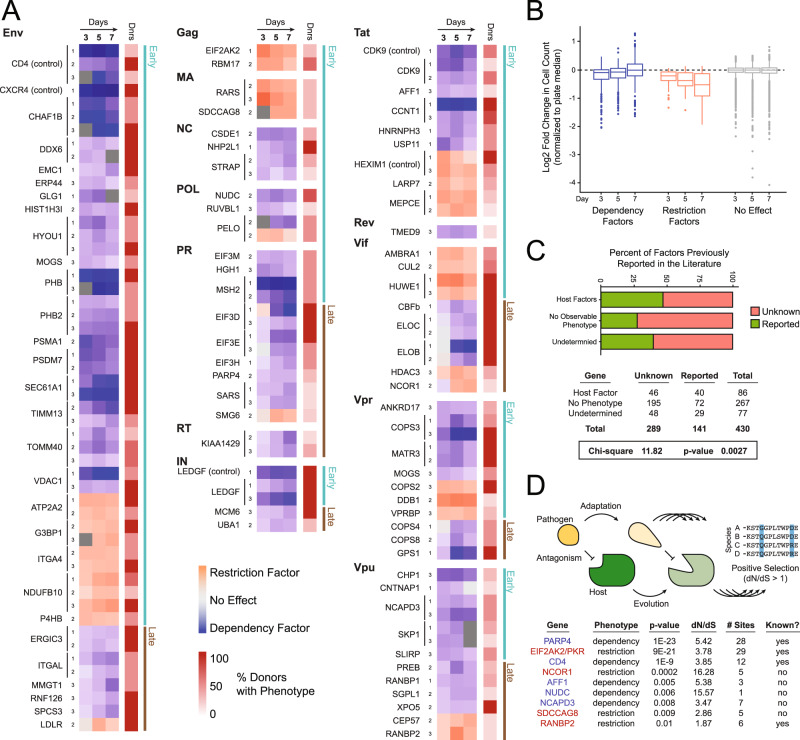
Identification of 86 candidate HIV host factors in primary CD4+ T cells. **A** Heatmap of the donor-average log_2_-normalized HIV-infection rate at each timepoint for each gRNA called as a hit (purple = decreased infection, pink = increased infection). Each gRNA is grouped by the HIV protein the target gene was found to interact with physically and by early- versus late-presenting phenotypes on the far left. Only donors that reached significance were included in the averages; the percent of donors showing the phenotype are indicated in an adjacent, red-colored heatmap. **B** Box-and-whisker plot of the distribution of log_2_-normalized cell counts per day for called dependency factors, restriction factors, and genes with no phenotype; center line, median; box limits, upper and lower quartiles; whiskers, 1.5x interquartile range; points, outliers. **C** Horizontal stacked bar chart representing the percent of factors previously reported to be HIV host factors in the NCBI GeneRIF database (green) per phenotypic designation, Chi-square test and *p*-value reported below. **D** Theoretical example of a host–pathogen arms race driving positive selection (top) with a table summarizing genes found under evolutionary positive selection in this study (bottom). The table summarizes the gene under selection (blue = candidate dependency factor, red = candidate restriction factor), the phenotype reported here, the ratio of nonsynonymous to synonymous changes in the gene body (dN/dS), *p*-value, number of sites under selective pressure, and if selection had been previously reported. A likelihood-ratio test was used to obtain a *p*-value, by comparing twice the difference in log-likelihoods with the chi-squared distribution with one degree of freedom; the Benjamini–Hochberg procedure was used to control the false-discovery rate.

Thresholds for candidate hit calling at each timepoint were defined empirically, such that fewer than 1% of gRNA with inefficient editing (i.e., mutational efficiency of less than 30%) had changes in infection beyond the threshold (0.6, 0.56, and 0.57 log_2_ fold change on days 3, 5, and 7, respectively, Fig. 2D, Supplementary Fig. 2b). For additional stringency, gRNA was required to exceed the threshold over two or more timepoints or across two or more donors. In other words, a gRNA had to have editing efficiency over 30% and resulted in a change in infection beyond the threshold at multiple timepoints or across multiple donors to be considered a hit. In total, 133 gRNA satisfied these criteria, implicating 90 genes (including the *CD4*, *CXCR4*, *LEDGF*, and *HEXIM1* controls) as potential HIV-dependency or restriction factors in primary CD4+ T cells (Fig. 3A, Supplementary Data 2). Of these, 40 genes yielded significant infection phenotypes across all donors analyzed, while the other 50 hits showed some donor dependency (Fig. 3A, Supplementary Data 3).

Of the 435 protein–protein interactors in the original report^23^, these combined experimental and computational analyses revealed 86 candidate HIV host factors that influence replication of HIV-1 NL4–3 in CD4+ T cells. Twenty-three genes yielded a restriction-factor phenotype, increasing HIV replication upon knock out, while 62 genes yielded a dependency-factor phenotype, decreasing HIV replication upon knockout; one gene, PELO, yielded conflicting phenotypes dependent on donor (Fig. 3A, Supplementary Fig. 4a, Supplementary Data 3). In total, 269 genes yielded no observable HIV phenotype, despite sequence-confirmed gene editing of at least 50% of alleles, thus indicating that these genes likely do not have a functional role in HIV replication in activated primary CD4+ T cells ex vivo. An additional 80 genes remain functionally ambiguous due to an inability to specifically target them for knockout, low cell viability upon knockout, or insufficient editing (Supplementary Data 3).

### Initial characterization of candidate host factors

Knockout of host factors that influence early events in the HIV-replication cycle (entry, reverse transcription, uncoating, integration, and transcription) should influence the first round of replication and their effects should be apparent even at the first timepoint (day 3). Knockout of host factors that influence late events or compromise fitness of progeny virions (translation, assembly, budding, and maturation) should become increasingly apparent at later timepoints after multiple rounds of replication (days 5 and/or 7). We found that a majority of identified host factors elicited significant differences in replication at the first timepoint (58 genes, green bars), consistent with potential roles in the early lifecycle, while a minority only showed significant differences at later timepoints (28 genes, brown bars) (Fig. 3A, Supplementary Fig. 4c). Notably, host factors that physically interact with the viral accessory proteins (Vif, Vpr, and Vpu) and the viral protease (PR) were enriched in host factors with late phenotypes, consistent with their roles later in the viral lifecycle^5,9^. By contrast, a majority of host factors that bind to the processed HIV polyproteins, structural proteins, and the regulatory proteins Tat and Rev yielded earlier phenotypes^7,8^ (Fig. 3A, Supplementary Fig. 4c).

Among candidate dependency factors, we observed an increase in cell count over time, consistent with protection from the cytopathic effects of ongoing viral infection (Fig. 3B). Conversely, we observed a decrease in cell count over time upon restriction factor knock out, consistent with increased viral infection and increased cell death (Fig. 3B). Factors without a phenotype, but with efficient editing, led to no significant change in cell count over time. Of the 86 candidate host factors, 40 have been previously linked to HIV infection in the NCBI Gene References into Function (GeneRIF) database, illustrating the power of this approach for recapitulating known biology (Fig. 3C). Conversely, 46 of these factors have no previously reported role in HIV infection and several represent potential druggable targets^53^ (Fig. 3C, Supplementary Fig. 4b, Supplementary Data 4). Comparison of this dataset to those from previously published RNAi screens, however, revealed minimal overlap, perhaps reflecting the significant variation in the cell types used and in the gene-perturbation strategies employed (Supplementary Fig. 5). Collectively, these data demonstrate the ability of this arrayed proteomics-to-genetics approach to identify and functionally categorize host factors directly in primary cells.

Some host-restriction and dependency factors are known to be in evolutionary arms races with viral factors, as the conflicting interests of the host and virus in establishing or escaping interactions drive recurrent rounds of adaptation and counter-adaptation at protein–protein interaction interfaces^54^ (Fig. 3D). These arms races can result in numerous amino acid sequence changes over evolutionary time in a process known as positive selection, which can be detected in DNA-sequence alignments if the rate of nonsynonymous changes is higher than the rate of synonymous changes. We looked for evidence of positive selection in the coding regions of the 90 previously described host factors and novel candidates examined here, comparing the human amino acid sequences to at least 17 primate orthologs. By this method, we observed positive selection in nine genes (q-value threshold <0.05), of which four were already known to experience rapid evolution (CD4, RANBP2, EIF2AK2/PKR, and PARP4; Fig. 3D, Supplementary Data 5). CD4 and RANBP2, in particular, have already been shown to be in evolutionary arms races with HIV and other lentiviruses^55–57^, while EIF2AK2/PKR restricts many viruses and its rapid evolution could be driven by any or all of these pathogens^58^. We previously described rapid evolution of dependency factor PARP4^59^, but the competing entity driving that evolution has not yet been identified. We also find novel evidence for positive selection in five additional genes: restriction factors NCOR1 and SDCCAG8, and dependency factors AFF1, NCAPD3, and NUDC.

### Vif- and Tat-binding host factors

**Fig. 4:**
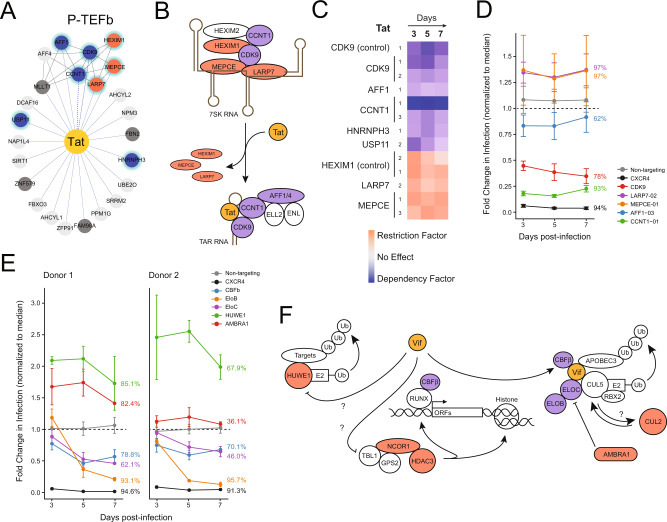
A functional map of HIV Tat and Vif complexes. **A** HIV Tat (yellow) is connected by blue edges to protein–protein interactors; black edges connect known human complexes (Corum database)^23,77^. Candidate or known dependency and restriction factors are annotated with blue and red nodes, respectively; all Tat interactors have early-acting phenotypes and thus are annotated with green halos. Factors determined to have no functional role in HIV infection are light gray, while those of undetermined phenotype are in dark gray. **B** A schematic model of superelongation complex formation by HIV Tat. Host factors with identified dependency or restriction factor phenotypes in the study are colored blue or red, respectively. **C** Heatmap of the donor-average log_2_-normalized HIV-infection rate at each timepoint for each gRNA called as a hit (purple = decreased infection, pink = increased infection), focused on Tat-interacting factors. **D** Fold change in HIV infection upon knockout of a subset of Tat interactors. Points represent an average of three technical replicates in cells from two donors ±SD. Average mutational efficiency of each guide across donors is annotated at the right of each line. **E** Fold change in HIV infection upon knockout of a subset of Vif interactors. Points represent an average of three technical replicates ±SD for two donors plotted independently. The mutational efficiency of each guide is annotated at the right of each line. **F** Schematic depicting putative functions of known and novel HIV Vif-interacting PPIs. Shading indicates dependency (blue) or restriction factors (red) in this study.

HIV Tat is required to promote transcriptional elongation of proviral transcripts by recruitment of the P-TEFb complex (composed of host proteins CDK9 and CCNT1) to the TAR stemloop and the subsequent assembly of the super elongation complex (including AFF1, AFF4, ENL, and ELL2)^60^. P-TEFb can alternatively be held in an inactive state by sequestration in the 7SK RNA complex composed of 7SK RNA, MEPCE, LARP7, and HEXIM1^33,42^. Consistent with these described roles, MEPCE, LARP7, and HEXIM1 were all found to act as restriction factors, while CDK9, CCNT1, and AFF1 were all found to act as dependency factors in primary T cells. All of these factors act early in the replication cycle and yield significant phenotypes at day 3 (Figs. 3A,  4A–D). Of the Tat-interacting hits, only the splicing factor HNRNPH3^61^ and the deubiquitinase USP11^62^ have not previously been linked to HIV replication, though these data suggest potential roles in HIV transcription.

Vif, an accessory protein, recruits an E3 ubiquitin ligase complex composed of CUL5, ELOB, ELOC, CBFβ, and RBX2 to degrade the antiviral APOBEC3 restriction factors, which otherwise package into virions and inhibit faithful reverse transcription during subsequent rounds of infection^29–31,51^. While editing of CUL5 failed to reach the 30% threshold required for phenotype calling, ELOB, ELOC, and CBFβ were all found to act as dependency factors in this study (Fig. 3A). Consistent with their known roles late in the replication cycle, this phenotype was more pronounced at days 5 and 7 compared with day 3 (Fig. 4E). Interestingly, we also found several potential restriction factors among the Vif PPIs, including HUWE1, AMBRA1, HDAC3, NCOR1, and CUL2 (Figs. 3A,  4E–F). We recently demonstrated that AMBRA1, a known DDB1- and CUL4-associated factor (DCAF), associates with the CUL4A complex and targets ELOC for ubiquitination and degradation^34^. HUWE1 and CUL2 are involved in other ubiquitin-ligase complexes but their connection to Vif remains unknown. The other two restriction factors associated with Vif, HDAC3 and NCOR1, form part of a histone-deacetylation complex that has been previously implicated in regulation of the proviral promoter^63^. Future work will be required to further characterize these and other novel HIV host factors, though the physical and functional handles described here should hasten these studies.

### Functional network mapping of HIV-host interactions

**Fig. 5:**
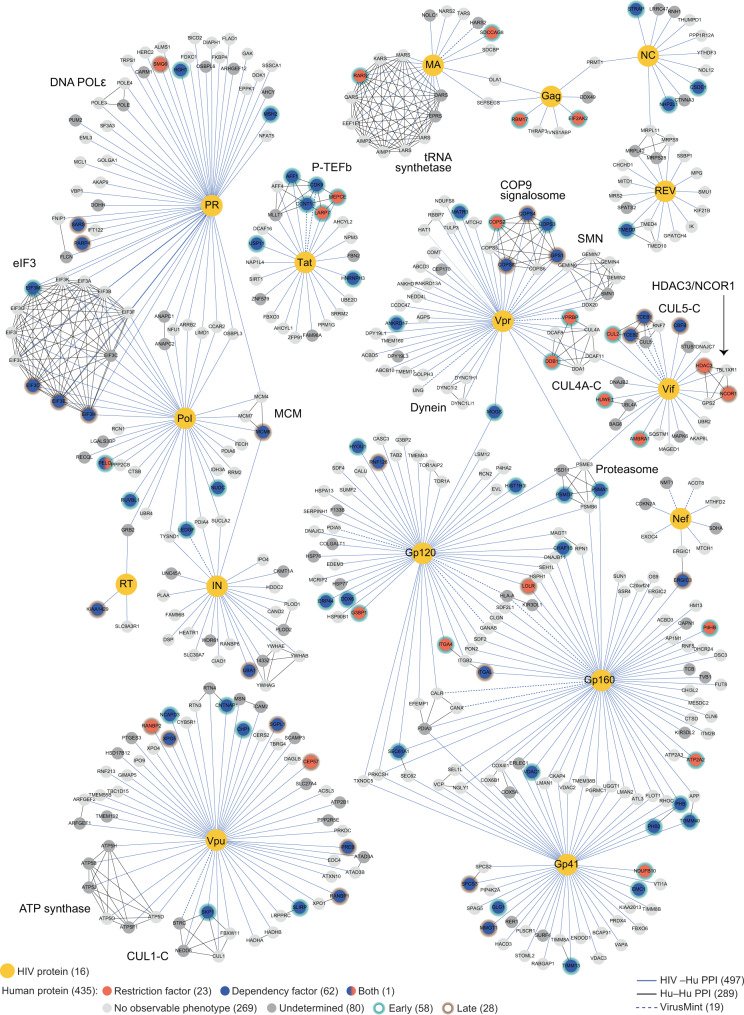
A functional map of HIV-host interactions. HIV proteins (yellow) are connected by blue edges to protein–protein interactors; black edges connect known human complexes (Corum database)^77^; dotted lines represent annotated HIV-host PPIs in the VirusMint database^78^. Candidate dependency and restriction factors are represented as blue and red nodes, respectively. Candidate host factors with early-acting or late-acting phenotypes have green or brown halos, respectively. Factors with no functional role in HIV infection observed here are light gray, while those of undetermined phenotype are dark gray. Figure is adapted from^23^.

Overlaying these genetic data onto the biochemical HIV-host-interaction map reveals a functional map of HIV-host complexes in primary human CD4+ T cells (Fig. 5). Overall, 19.8% of identified PPIs were identified as candidate host factors in primary CD4+ T cells. Excluding genes that could not be knocked out (and so remain functionally ambiguous), this implies that up to 24.2% of the physical network may have a functional role in this model system. Previously published genome-wide screens using arrayed RNAi or pooled CRISPR approaches have identified host factors at a rate of 0.1–2% of the starting pool^13,15–17,19,20^, suggesting a strong enrichment in host-factor identification when starting with a proteomic interactome dataset. While protein–protein-interaction score did not correlate with host-factor validation (Supplementary Fig. 6a), fewer restriction factors were identified as PPIs in HEK293T cells when compared with Jurkat cells (Supplementary Fig. 6b), emphasizing the need for high quality interactome data collected in physiologically relevant cell models.

### Validation of host factors

**Fig. 6:**
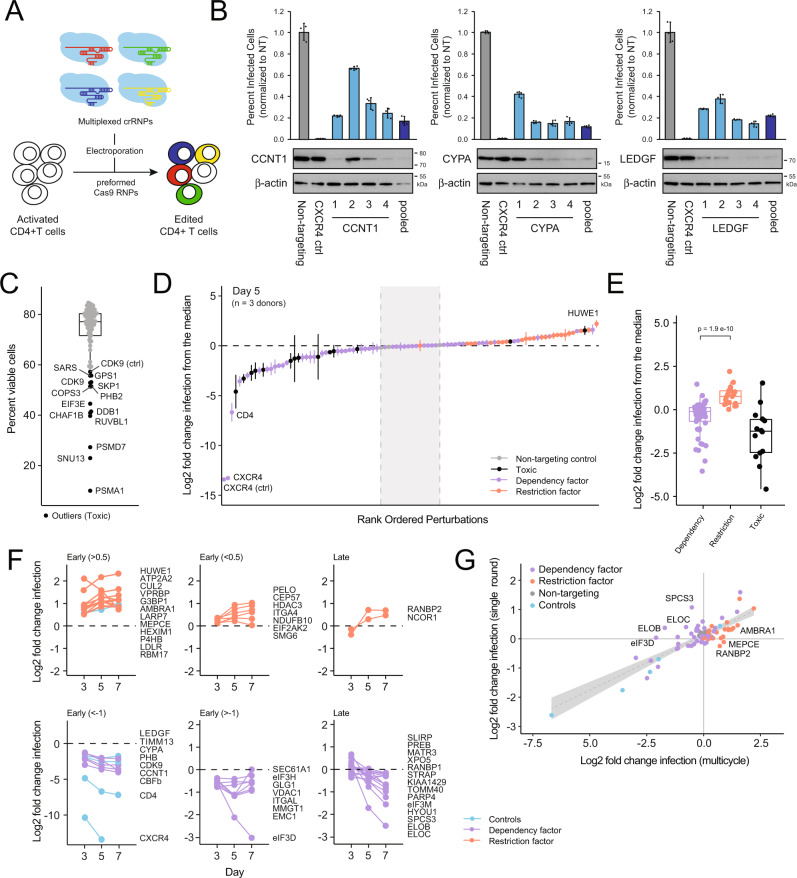
Hit validation using multiplexed gRNA. **A** Schematic of the multiplex gRNA approach for gene knock out in primary CD4+ T cells. **B** Bar charts depicting percent HIV-infected cells post challenge upon knockout with individual gRNA 1 through 4 versus a multiplexed pool of gRNAs 1 through 4 (average of technical triplicates ±SD). Western blots below depict protein depletion for each targeted gene. Three independent loci were targeted: *CCNT1* (left), *CYPA* (middle), and *LEDGF* (right). **C** Box-and-whisker plot of the average percent of live CD4 + T cells in each well four days after electroporation with multiplexed gRNA; center line, median; box limits, upper and lower quartiles; whiskers, 1.5x interquartile range; black points, outliers. Outlier points are considered toxic and are labeled by targeted gene name. **D** S-curve plot of the log_2_ fold change in infection relative to the plate median at day 5 for each multiplexed gRNA, averaged across all 3 donors ±SD. The dashed black line indicates the median; the dashed gray lines represent the nontargeting range. Dots are colored by toxicity and by phenotype in the original screen as indicated. **E** Box-and-whisker plot of the distribution of log_2_-normalized HIV-infection rates for dependency factors (*n* = 52) versus restriction factors (*n* = 21) versus essential genes (*n* = 13) at day 5. Center line, median; box limits, upper and lower quartiles; whiskers, 1.5x interquartile range; *p*-value reflects a two-sided Wilcoxon rank-sum test comparing dependency factors and restriction factors each measured in three biologically independent replicates. **F** Line chart of log_2_-normalized HIV-infection rates over time for each validated hit (two-sided Wilcoxon rank-sum test, *p*-value < 0.1 at multiple timepoints). Restriction factors are shown above and dependency factors shown below with relevant controls. Genes with significant differences at day 3 are coded “early” and sorted by magnitude of effect; genes with significant differences at only days 5 or 7 are coded as “late”. **G** Log_2_-normalized HIV-infection rates at day 5 after multicycle replication versus at day 3 after single-cycle replication in the presence of Saquinavir. The linear regression line with 95% confidence interval is shown in gray.

To validate the host-factor candidates reported here, we repeated these experiments using a new panel of gRNA in cells from three independent human-blood donors. Rather than using an array of individual gRNA for each gene, four different gRNA per gene were multiplexed into a single well to generate multiplexed Cas9 RNPs (Fig. 6A). To confirm this approach worked, we first compared the efficacy of multiplexed Cas9 RNPs to the efficacy of RNPs containing each constituent gRNA at three independent loci encoding the well-described HIV host factors *CCNT1*, *CYPA*, and *LEDGF*. Four days after electroporation, protein lysates were collected for Western blotting and cells were challenged with HIV-1 NL4–3 nef:IRES:GFP in technical triplicate as above. Percent infected cells were quantified by flow cytometry three days post challenge. Consistent with our deep-sequencing results, we observed variability in the efficiency of each individual gRNA at the protein level (Fig. 6B). However, the multiplexed pool of gRNA resulted in consistent protein depletion similar to the best gRNA contained in the pool. Furthermore, the percent of HIV-infected CD4+ T cells was significantly decreased by each pool relative to the nontargeting control similar to the degree observed with the most efficient gRNA in the pool (Fig. 6B). Taken together, these results suggest that gRNA multiplexing may be a viable and cost-effective way to minimize arrayed screening—especially for validation and characterization of selected hits—without sacrificing overall efficacy.

Taking advantage of this approach, multiplexed RNPs were generated for all 86 candidate host factors identified above, as well as for four control genes: *CD4, CXCR4, LEDGF*, and *HEXIM1*. As before, these were delivered to T cells from three independent blood donors by electroporation alongside the 6 individual, previously validated control RNPs: three with nontargeting negative-control gRNA and three with positive-control gRNA targeting *CXCR4*, *LEDGF*, and *CDK9*. Viability was monitored by amine-dye staining and flow cytometry four days post electroporation. While a majority of perturbations had no impact on cell viability across the three donors, 13 were statistical outliers, significantly decreasing viable cell counts (Fig. 6C). Both the multiplexed and single gRNA knockout of CDK9 yielded similar viability defects by this measure. Cells were subsequently split six ways and challenged with HIV-1 NL4–3 nef:IRES:GFP. Half of the plates were treated with the protease inhibitor saquinavir (SQV) 24 h after addition of virus to limit replication to a single round of infection. Spreading infection was monitored at days 3, 5, and 7 by flow cytometry, whereas single-round infection was monitored at day 3 only. The log_2_ fold change was calculated relative to the plate median and averaged across donors.

Overall, these results were in agreement with those obtained in the original screen. Relative HIV-infection rates (average log_2_ fold change) differed significantly between the previously called dependency and restriction factors as expected (Fig. 6E) with the nontargeting gRNA all distributed tightly near the plate median (Fig. 6D). Focusing on the day-5 timepoint, 38 of the targeted genes decreased HIV infection beyond the range of the nontargeting gRNAs. These included the CD4, CXCR4, LEDGF, and CDK9 control guides as well as 32 of the 62 dependency factors called in the original screen. Of note, 11 of these factors resulted in viability defects, which could confound these results (Fig. 6C). On the other side, 38 of the targeted genes increased HIV infection beyond the range of the nontargeting gRNAs (Fig. 6D). These included the HEXIM1 control guide, PELO, and 22 of the 23 originally identified restriction factors. While a number of previously called dependency factors also increased infection here, they largely clustered near the nontargeting guides and likely reflect some bias caused by normalization to the plate median, given the enrichment of putative dependency factors (Fig. 6D). Overall, these data confirmed 55 of the 86 hits originally called in the screen in independent knockout experiments across three independent human-blood donors for a 64% confirmation rate.

Assuming that each host factor was edited to the same extent and would have the same relative magnitude of impact on HIV replication in cells from each individual donor, we can treat each donor as a biological replicate and calculate significance using a Wilcoxon rank sum test. These differences should persist over the time course of infection such that significant differences are apparent over at least two timepoints with the exception of those genes that only reach significance at day 7. By this more stringent metric (Wilcoxon rank sum, adjusted *p*-value < 0.1 over at least 2 timepoints or at day 7), 47 of the 86 hits originally called in the screen recapitulated their phenotype (55%, Fig. 6F), while 13 showed toxicity (15%), and 26 (30%) did not confirm. The multiplex nature of the approach precluded simple amplicon sequencing to calculate knockout efficiency, so it is unclear how many of these failed to recapitulate due to a lack of editing versus a lack of a phenotype.

As before, we see several different trends in the replication profiles upon gene knockout. Some genes result in strong effects on replication even early in the infection timecourse (by day 3), while others have significant impacts only at later timepoints (Fig. 6F). Delayed phenotypes could either be due to genes only having an impact during the late stages of viral replication or due to small magnitude changes building over multiple rounds of infection. To differentiate between these possibilities, we directly compared the spreading infection data collected at day 5 with the single-round data collected at day 3 in the presence of SQV (Fig. 6G). A majority of genes fell along a steady diagonal, with a log_2_ fold change of 1 in single-cycle roughly equating to a log_2_ fold change of 3 in multicycle replication. These factors all likely act during the early stage of viral replication. However, a subset of dependency factors only yielded phenotypes in multicycle replication and fell above the diagonal, including the well-known Vif-interacting factors ELOB and ELOC, as well as the novel hits SPCS3 and EIF3D (Fig. 6G). Likewise, a handful of restriction factors only yielded phenotypes in multicycle replication and so fell below the diagonal, including the Vif-interacting factor AMBRA1^34^. These phenotypes are consistent with roles in the late stage of replication. Ultimately, these data will help in the mechanistic characterization of the newly described host factors reported here.

## Discussion

By leveraging a proteomics-to-genetics platform, our study identified 86 candidate HIV host factors in primary CD4+ T cells, 47 of which were validated in follow-up assays. Altogether, over 10% of protein interactors validated as functional host factors, a significant enrichment over identification rates in genome-wide screens^13,15–17,19,20^ (Table 1). Of the initial candidates, 40 genes were already associated with HIV replication in the literature, demonstrating the power of the approach to uncover real biological features of pathogens. An additional 46 new candidate host factors were nominated and systematically tested for cell-viability effects, positive-selection signatures, and early versus late lifecycle effects. Ultimately, we hope these results will serve as a significant resource for deciphering the molecular mechanism of these factors and as a roadmap for host-factor discovery in emerging and understudied pathogens.

While these results aligned well with the published literature, they correlated poorly with results from previous arrayed or pooled genome-wide RNAi screens in cell lines, reflecting the unique biology of the different model systems used and underlining the importance of studying therapeutically relevant primary cells^14,18^ (Supplementary Fig. 5). Expanding this approach to other primary targets of HIV, notably tissue-resident cells and myeloid populations, will likely reveal important roles for additional host factors^64^. We believe this principle extends far beyond studies of HIV or host–pathogen interactions. Advances in gene editing and other technologies should make primary human cells a principal system of investigation for discovery- and hypothesis-based inquiries in future biomedical research, rather than solely a confirmatory or validating model system.

Importantly, our arrayed screen approach provided paired editing efficiency and HIV-infectivity data. This not only validates the approach, as we see the frequency and magnitude of changes in HIV-phenotype increase as editing increases, but also allows for the disambiguation of negative results. While roughly 80% of total gene targets yielded no infectivity phenotype, three-quarters of these were efficiently targeted for knockout, implying that these factors have no functional role in infection in the context of our assay (Fig. 2D). These factors may only be critical for replication of other HIV-1 strains or under other in vitro or in vivo conditions, may have functional roles in other cell types not assayed here, may be false positives in the proteomic data, or may be functionally redundant and therefore require systematic double-knockout studies to reveal their phenotypes^65^.

Despite the strength of this approach, it also has a number of potential drawbacks. First, the reliance on a physical interactome dataset introduces a bias toward identification of factors that are amenable to detection by affinity-purification mass spectrometry, most notably factors that exist in stable, soluble complexes. These studies are furthermore typically conducted in cell-line models that do not fully recapitulate the physical networks that may exist in primary cell types, potentially complicating interpretation of the results. Second, limitations in cell numbers necessitated the use of cells from several independent donors that may or may not be directly comparable in editing efficiency, basal susceptibility to HIV-1 infection, or magnitude of phenotypic impact after knockout. We attempted to account for these limitations by normalizing data within donors, benchmarking set controls, and relying on dual thresholds of editing efficiency and infection-rate change. Nevertheless, in separate validation experiments leveraging a different approach to gene editing and statistical hit calling, 30% of candidates failed to recapitulate, emphasizing the importance of secondary validation.

The adoption of interdisciplinary approaches, such as the proteomics-to-genetics strategy taken here, will be critical to streamline experimental-discovery pipelines, more quickly validate novel drug targets, and enhance translational research^66–69^. While current combination antiretroviral-therapy regimens are triumphs of biomedical science that have changed the face of the HIV epidemic^2^, there is increasing recognition of the morbidity and mortality costs associated with failure to clear the virus and long-term use of these drugs, motivating a search for alternative treatment modalities. This functional map of HIV host factors in primary cells provides several leads for future functional interrogation and will hopefully open the door to additional therapeutics that physically antagonize virus–host protein–protein interactions^10–12^.

## Methods

### Plasmid constructs

Replication-competent reporter-virus stocks were generated from an HIV-1 NL4–3 molecular clone, wherein GFP has been cloned behind an internal ribosomal entry site (IRES) cassette following the viral *nef* gene^49^ (AIDS Reagent Program #11349).

### Cell lines

HEK293T cells (ATCC, CRL-3216) used for the production of HIV-1 virus and HeLa–TZM cells used for titering supernatants^70^ (AIDS Reagent Program #1470) were maintained in Dulbecco’s modified Eagle’s medium (Corning or Gibco) with 10% fetal bovine serum (FBS, Gibco) and 25 μg/mL penicillin/streptomycin (P/S, Corning or Gibco) in humidified atmosphere at 37 °C/5% CO_2_.

### Primary CD4+ T cell isolation and culture

Detailed protocols for primary CD4+ T-cell isolation and culture can be found here^36^. Briefly, primary human T cells were isolated from healthy human donors either from fresh whole blood obtained after informed consent under a protocol approved by the UCSF Committee on Human Research (CHR #13-11950), or from leukoreduction chambers after Trima apheresis (Blood Centers of the Pacific, now Vitalant). Peripheral blood mononuclear cells (PBMCs) were isolated by Ficoll centrifugation using SepMate tubes (STEMCELL, per manufacturer’s instructions). T cells were subsequently isolated from PBMCs by magnetic negative selection using an EasySep Human T Cell Isolation Kit (STEMCELL, per manufacturer’s instructions).

Isolated CD4^+^ T cells were suspended in complete Roswell Park Memorial Institute (RPMI) media, consisting of RPMI-1640 (Sigma) supplemented with 5 mM 4-(2-hydroxyethyl)-1-piperazineethanesulfonic acid (HEPES, Corning), 2 mM glutamine (UCSF Cell Culture Facility), 50 μg/mL penicillin/streptomycin (P/S, Corning), 5 mM sodium pyruvate (Corning), and 10% fetal bovine serum (FBS, Gibco). Media was supplemented with 20 IU/mL IL-2 (Miltenyi) immediately before use. Cells were immediately stimulated on anti-CD3-coated plates [coated for 2 h at 37 °C with 20 µg/mL anti-CD3 (UCHT1, Tonbo Biosciences)] in the presence of 5 µg/mL soluble anti-CD28 (CD28.2, Tonbo Biosciences). Cells were stimulated for 72 h at 37 °C/5% CO_2_ prior to electroporation.

### RNP production

Detailed protocols for RNP production and T-cell editing have been previously published^36^. Briefly, lyophilized crRNA and tracrRNA (Dharmacon) was resuspended at a concentration of 160 µM in 10 mM Tris-HCL (7.4 pH) with 150 mM KCl. Cas9 ribonucleoproteins (RNPs) were made by incubating 5 µL of 160 µM crRNA with 5 µL of 160 µM tracrRNA for 30 min at 37 °C, followed by incubation of this 80 µM gRNA:tracrRNA complex product with 10 µL of 40 µM Cas9 (UC Berkeley Macrolab) to form RNPs at 20 µM. Five 3.5 µL aliquots were frozen in Lo-Bind 96-well V-bottom plates (E&K Scientific) at −80 °C until use. All crRNA guide sequences were from the Dharmacon predesigned Edit-R library for gene knockout. For synthesis of multiplexed RNPs, four independent crRNA targeting the same gene were mixed at a 1:1:1:1 ratio prior to addition of the tracrRNA as above.

### T cell editing

Detailed protocols for RNP production and T-cell editing have been previously published^36^. Briefly, after three days of stimulation as above, cells were suspended and counted. Each reaction consisted of 4 × 10^5^ cells, 3.5 µL of RNP, and 20 µL of electroporation buffer. Immediately before electroporation, cells were centrifuged at 400 × *g* for 5 min, the supernatant was removed by aspiration, and the pellet was resuspended in 20 µL of room-temperature P3 electroporation buffer (Lonza) per reaction. The cell suspension was then gently mixed with thawed RNP and aliquoted into a 96-well electroporation cuvette for nucleofection with the 4D 96-well shuttle unit (Lonza) using pulse code EH-115. Immediately after electroporation, 80 µL of prewarmed media without IL-2 was added to each well and cells were allowed to rest for at least one hour in a 37 °C cell culture incubator. Subsequently, cells were moved to 96-well flat-bottom culture plates prefilled with 100 µL of warm complete media with IL-2 at 40 IU/mL (for a final concentration of 20 IU/mL) and anti-CD3/anti-CD2/anti-CD28 beads (T cell Activation and Stimulation Kit, Miltenyi) at a 1:1 bead:cell ratio.

Cells were cultured at 37 °C/5% CO_2_ in a dark, humidified cell culture incubator for a further 6 days to allow for gene knockout, with media supplementation on days 3 and 5. On day 6, one-eighth of each culture, approximately 35 µL, was removed for the extraction of genomic DNA and subsequent mutational analysis by deep sequencing. Cells were mixed at a 1:1 vol:vol ratio with QuickExtract buffer (EpiCentre) in a 96-well PCR plate. Plates were sealed with adhesive foil and heated at 65 °C for 20 min followed by 98 °C for 5 min. Genomic DNA extracts were stored at −20 °C until use. A further 35 µL of culture was reserved for protein lysates. Cells were pelleted, supernatant was removed, and pellets were resuspended in 70 µL of 2.5x Laemmli Sample Buffer. Protein lysates were heated to 98 °C for 20 min before storage at −80 °C for later use.

### Preparation of HIV stocks

Replication-competent reporter-virus stocks were generated from an HIV-1 NL4-3 molecular clone wherein GFP had been cloned behind an internal ribosomal entry site (IRES) cassette following the viral *nef* gene. Briefly, 10 µg of molecular clone was transfected (PolyJet, SignaGen) into 5 × 10^6^ HEK293T cells (ATCC CRL-3216) according to the manufacturer’s protocol. In all, 25 mL of supernatant was collected at 48 and 72 h and combined. Virus-containing supernatant was filtered through 0.45 mm polyvinylidene fluoride (PVDF) filters (Millipore) and precipitated in 8.5% polyethylene glycol (PEG, average M_n_ 6000, Sigma), 0.3 M sodium chloride for 4 h at 4 °C. Supernatants were centrifuged at 3500 rpm for 20 min and virus resuspended in 0.5 mL of phosphate-buffered saline (PBS) for a 100x effective concentration. Aliquots were stored at −80 °C until use.

### HIV infection

Detailed protocols for HIV-spreading infection have been previously described^36^. Briefly, 6 days post electroporation, cells were replica-plated into triplicate 96-well round-bottom plates and cultured overnight in 150 µL of complete RPMI as described above in the constant presence of 20 IU/mL IL-2. On the following day, 2.5 µL of concentrated virus was added to each well in a 50 µL carrier volume to bring the total volume in each well to 200 µL. Cells were cultured in a dark, humidified incubator at 37 °C/5% CO_2_. On days 3 and 5 post infection, 75 µL of each culture was removed and mixed 1:1 with freshly made 2% formaldehyde in PBS (Sigma) and stored at 4 °C for analysis by flow cytometry. Cultures were supplemented with 75 µL of complete, IL-2-containing RPMI media and returned to the incubator. On day 7 post infection, 150 µL of culture was sampled and mixed with 50 µL of freshly made 4% formaldehyde solution for a final concentration of 1% formaldehyde and stored at 4 °C for analysis by flow cytometry. The remaining cultures were bleached and discarded per institutional biosafety regulations. For single-round infection assays, each well is supplemented with Saquinavir to a final concentration of 5 µM 24 h post challenge. On day 3 post infection, 75 µL of each culture was removed and mixed 1:1 with freshly made 2% formaldehyde in PBS (Sigma) and stored at 4 °C for analysis by flow cytometry.

### Flow cytometry and analysis of infection data

Flow-cytometric analysis was performed on an Attune NxT Acoustic Focusing Cytometer (ThermoFisher), recording all events in a 100 µL sample volume after one 150 µL mixing cycle. Data were exported as FCS3.0 files using Attune NxT Software v3.2.0 and analyzed with a consistent template on FlowJo v10.5.3. See Supplementary Fig. 2 for the gating strategy and representative results.

### PCR amplification of cut sites

Sequencing was conducted as previously described^40^. PCR primers were designed using a Python wrapper around Primer3 (github.com/czbiohub/Primer3Wrapper) (Leenay et al., 2018). This pipeline was used to design 180 to 260 nucleotide amplicons, ensuring that the cut site was at least 50 nucleotides from the end of each primer, as well as 15 nucleotides from the center of the read. Sequencing adapters (forward: 5′-CTC TTT CCC TAC ACG ACG CTC TTC CGA TCT-3′ and reverse 5′-CTG GAG TTC AGA CGT GTG CTC TTC CGA TCT-3′) were appended to the designed primers. Sites were amplified using between 4000 and 10,000 genomic copies, 0.5 µM of each primer, and Q5 hot-start high-fidelity 2x master mix (NEB). PCR was performed using a standard protocol: 98 °C for 30 seconds; then 35 cycles of 98 °C for 10 seconds, 60 °C for 30 seconds, and 72 °C for 30 seconds; followed by a final extension at 72 °C for 2 min. Samples were diluted 1:100 and individually indexed in a second, 12-cycle PCR using index primers containing Illumina sequencing adapters and eight base barcodes, under the same conditions as the first PCR. After the second PCR, indexed samples were pooled and purified using a 0.7x SPRIselect purification and sequenced on an Illumina NextSeq 500.

### Analysis of deep sequencing data

Raw sequencing files were filtered, trimmed and aligned as previously described^40^. Each sample was individually analyzed using the CrispRVariants Bioconductor (Release 3.14) package in R^71^, which performs a secondary alignment and quantifies each unique insertion and deletion per sequencing read. Repair outcomes were then further parsed using embedded CrispRVariants packages to quantify mutational efficiencies as the fraction of reads with frameshift or an insertion or deletion greater than two amino acids.

### Analysis and filtering of infection data

Flow data were analyzed with a standard template in FlowJo (v10.5.3, TreeStar) (refer to Fig. S2 for gating strategy), and data were exported to .csv files. These files were then imported into R using RStudio (v1.4). Wells with too few lymphocytes (<1000) or nonautofluorescent singlets (<500) were excluded from analysis (Fig. S2). Log_2_ fold change in infection in each well was computed relative to the plate median. Edge correction was done assuming that the ratio of the median infection of a given edge to the median of the center of the plate should be consistent across biological and technical replicates. After normalizing edges to this factor, log_2_ fold changes were recalculated, and average and standard deviation of infection and cell count were computed for each set of technical triplicates. Samples with low average cell count (<−1.45 log_2_ fold change relative to plate median) or high variability (SD > 2 log_2_ fold change in infection or SD > 1.5 log_2_ fold change in cell count) across technical triplicates were redacted. Sequencing data were then imported and matched to individual wells. Per-day thresholds for significance were calculated by focusing on data with poor mutational efficiency (<30%) as representative of phenotypic noise. The thresholds were set by iteratively calculating the maximal log_2_ fold change in infection that still left fewer than 1% of points positive, defined as having an absolute value(L2FC) > threshold. From the list of all guides with significant points, significant genes were identified by having either: (1) significance in at least one point across greater than 50% of all donors tested with that guide, or (2) strong, donor-dependent phenotype of two or more timepoints significant in the same guide and same donor. Dependency and restriction factors were identified in separate one-sided analyses such that guides must have log_2_ fold change in the same direction across donors and timepoints to be called a hit. All raw and averaged infection data and cell counts from the initial screen are available in Supplementary Data 6 and 7, respectively. All raw and averaged infection data and cell counts from the multiplexed validation experiment screen are available in Supplementary Data 8.

### Literature review

An unbiased literature review of the 435 screened genes was performed to determine whether a functional role in HIV biology had been previously demonstrated. GeneRIFs were downloaded for all genes with annotated HIV relevance on December 22, 2017. A subsequent manual keyword search was conducted and completed on August 23, 2018. Each potential host factor was identified using NCBI GeneID and Uniprot accession number. All gene and protein aliases provided were searched in Google and Google Scholar using the identified gene or protein name or recognized aliases, and “HIV-1.” Further, literature cited in the NCBI HIV-1 interactions tab was reviewed for demonstration of a functional role. A gene was concluded to have a functional role only if demonstrated perturbation or inhibition of the gene product had been shown to positively or negatively alter HIV function. The results from previously described genome-wide HIV RNAi screens were not considered sufficient demonstration of the functional role for the purposes of this review. Refer to Supplementary Data 4.

### Immunoblotting

Cell lysates were prepared by suspension of cell pellets directly in 2.5x Laemmli Sample Buffer followed by homogenization at 98 °C for 30 min. Samples were run on 4–20% Tris-HCl SDS-PAGE gels (BioRad Criterion) at 90 V for 40 min followed by separation at 150 V for 70 min. Proteins were transferred to PVDF membranes by electrotransfer (BioRad Criterion Blotter) at 90 V for 2 h. Membranes were blocked in 4% milk in PBS, 0.1% Tween-20 for 1 h prior to primary antibody incubation overnight at 4 C. LEDGF (1:2000 dilution, clone C57G11, Cell Signaling Technologies, Cat. No. 2088 S), CCNT1 (1:1000 dilution, clone D1B6G, Cell Signaling Technologies, Cat. No. 81464 S), and CYPA (1:12000 dilution, polyclonal, Cell Signaling Technologies, Cat. No. 2175 S) levels were probed relative to β-actin (1:10000 dilution, clone 8H10D10, Cell Signaling Technologies, Cat. No. 3700 S) as a protein-loading control. Anti-rabbit or anti-mouse IgG horseradish peroxidase (HRP)-conjugated secondary antibodies (1:20000, polyclonal, Jackson ImmunoResearch Laboratories, Cat. Nos. 111-035-003 and 115-035-003) were detected using Pierce™ ECL Western Blotting Substrate (ThermoFisher). Blots were incubated in a 1xPBS, 0.2 M glycine, 1.0% SDS, 1.0% Tween-20, and pH 2.2 stripping buffer before reprobing. Refer to the **Source Data** file for full-blot scans.

### Positive selection analysis

For each gene, we obtained a human ORF sequence, choosing the splice isoform with the longest ORF. We used this ORF as query in a blastn search^72^ of NCBI’s NR database and for each nonhuman primate species, we collected the blast hit with the highest bit score, filtering out matches of <60% identity or <100 bp alignment length, and ignoring database sequences that are >20 kb long or have no annotated ORF. We also blasted each primate hit to a collection of all human genes, to ensure all sequences are reciprocal best hits (a proxy for true orthology, albeit imperfect). We extracted ORFs from each primate match, and aligned orthologous sequences using MACSE v2.00^73^, treating the human sequence as “reliable” and the other primate sequences as “less reliable” (parameters: -fs_lr 10 and -stop_lr 10). We then manually inspected and, if necessary, edited all alignments to remove unreliable sequence segments, as gene predictions found in NR sometimes contain erroneous exons. We used phyml v3.0^74^ to estimate a phylogeny for each alignment (parameters: -m GTR --pinv e --alpha e -f e). The alignment and phylogeny were then used as input for the codeml algorithm through PAML v4.9^75^, comparing the neutral/purifying model 8a (where dN/dS for codons follows a beta distribution with values between 0 and 1, with an extra class of sites with dN/dS fixed at 1) with model 8 that allows a subset of codons to have dN/dS > 1 (parameters: codon frequency F3x4, estimate kappa, initial kappa 2, initial omega 0.4, ncatG 10, and cleandata 0). We performed a likelihood-ratio test^75^ to obtain a p-value, by comparing twice the difference in log-likelihoods with the chi-squared distribution with 1 degree of freedom. After running all 88 analyses, we used the Benjamini–Hochberg procedure^76^ to control the false-discovery rate. We also used a custom script to remove codons in each alignment that overlap a CpG dinucleotide in any aligned species, and repeated PAML analysis as described above. All results from these analyses are reported in Supplementary Data 5.

### Reporting summary

Further information on research design is available in the Nature Research Reporting Summary linked to this article.

## Supplementary information

Supplementary Information

Description of Supplementary Files

Supplementary Data 1

Supplementary Data 2

Supplementary Data 3

Supplementary Data 4

Supplementary Data 5

Supplementary Data 6

Supplementary Data 7

Supplementary Data 8

Reporting Summary

## Data Availability

All raw sequencing data and downstream analyses are openly available through SRA (BioProject: PRJNA486372) and Figshare (10.6084/m9.figshare.6957119.v1)^40^. All raw and processed flow-cytometry data, mutational efficiency data, and gRNA sequences are provided here as Supplementary Data Files. All other data are available from the corresponding author upon reasonable request. Source data are provided with this paper.

## Source data

Source Data

## Code availability

All code used for the calculation of editing efficiency is available on FigShare as previously reported (10.6084/m9.figshare.6957119.v1)^40^. Furthermore, additional code generated for statistical calculations reported here has been uploaded to and is openly available on Figshare (10.6084/m9.figshare.7246652).
