# Supplementary material for: A functional map of HIV-host interactions in primary human T cells
Source: Nat Commun. 2022 Apr 1;13:1752. doi: 10.1038/s41467-022-29346-w (PMC8976027; doi:10.1038/s41467-022-29346-w)
Supplement: Supplementary file 1 — Supplementary Information [file 41467_2022_29346_MOESM1_ESM.pdf]

Supplementary Information:

A Functional Map of HIV-Host Interactions in Primary Human T cells

Hiatt *et al.*

Supplementary Files

1) Supplementary Figures and Figure Legends 1-6
2) Supplementary References

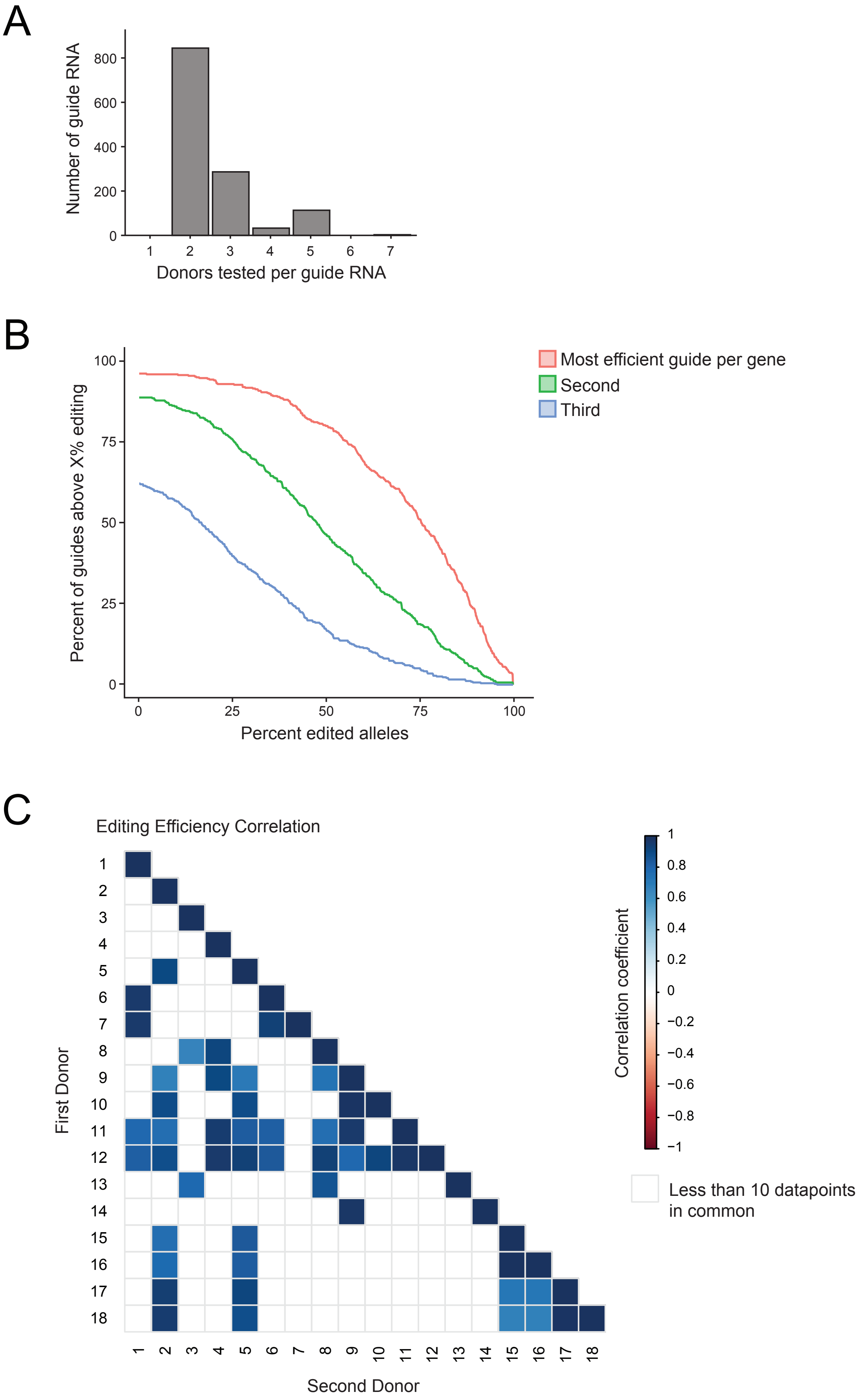
A
Number of guide RNA
800
600
400
200
0
1
2
3
4
5
6
7
Donors tested per guide RNA
B
Percent of guides above X% editing
100
75
50
25
0
Percent edited alleles
Most efficient guide per gene
Second
Third
C
Editing Efficiency Correlation
First Donor
Second Donor
1
2
3
4
5
6
7
8
9
10
11
12
13
14
15
16
17
18
Correlation coefficient
1
0.8
0.6
0.4
0.2
0
−0.2
−0.4
−0.6
−0.8
−1
Less than 10 datapoints in common

Supplementary Figure 1 | Mutational efficiency and donor-by-donor correlations. a, Histogram of the number of guide RNA tested in the number of unique donors, excluding control gRNA (median = 2 donors/gRNA, mean = 2.55/gRNA). **b**, Cumulative distribution function of editing efficiency for the most efficient, second most, and third most efficient guide against a given gene. Mutational efficiency was averaged across donors; guides without robust sequencing data were excluded from the plot. **c**, Heatmap of correlation of editing efficiency of guides between donors (minimum 10 guides in common, pairwise r^2 range 0.67-0.99, mean = 0.88).

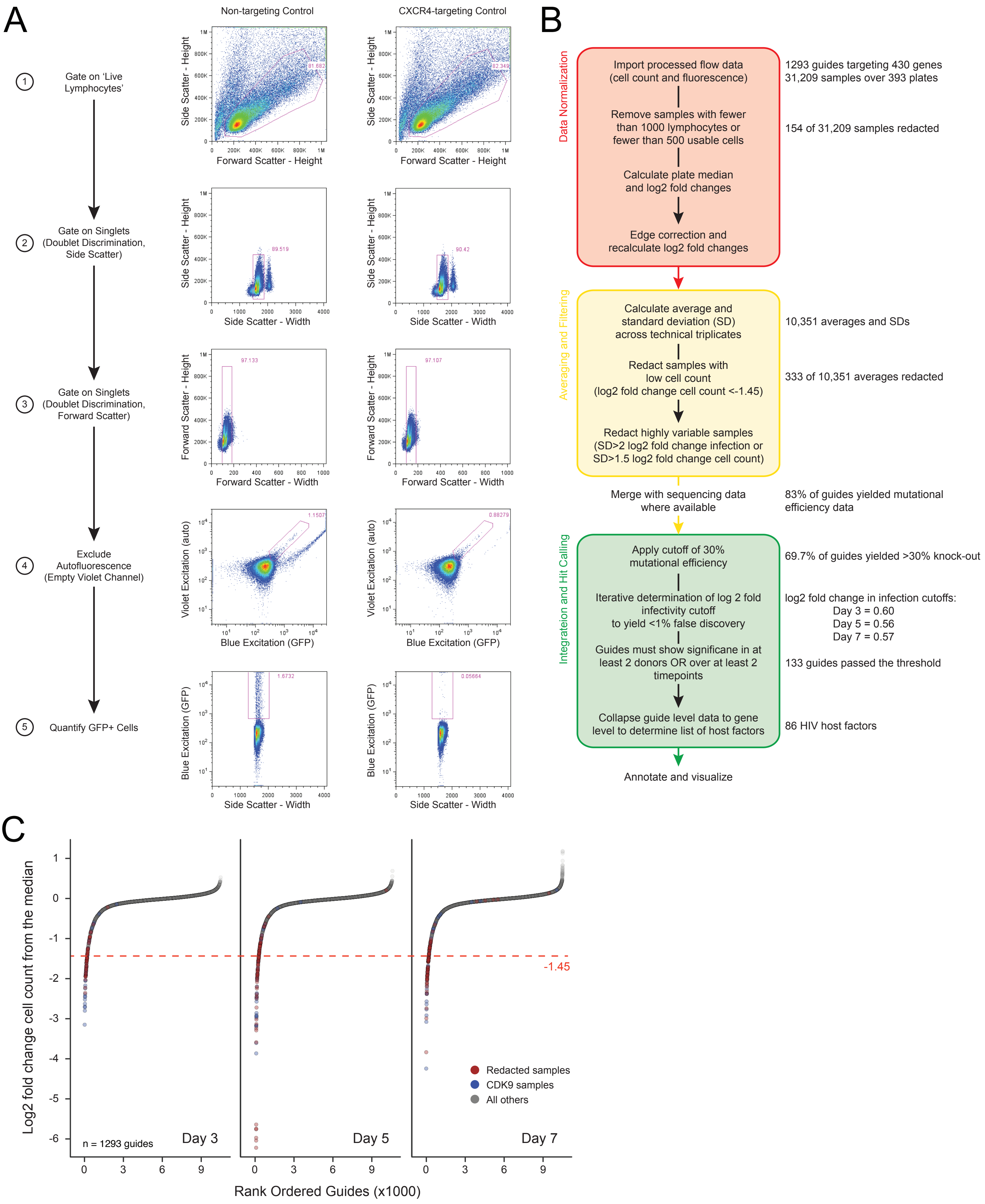
A
Non-targeting Control
CXCR4-targeting Control
1 Gate on 'Live Lymphocytes'
2 Gate on Singlets (Doublet Discrimination, Side Scatter)
3 Gate on Singlets (Doublet Discrimination, Forward Scatter)
4 Exclude Autofluorescence (Empty Violet Channel)
5 Quantify GFP+ Cells
Side Scatter - Height
Forward Scatter - Height
81.682
82.348
Side Scatter - Width
89.519
90.42
Forward Scatter - Height
Forward Scatter - Width
97.133
97.107
Violet Excitation (auto)
Blue Excitation (GFP)
1.1507
0.88279
Blue Excitation (GFP)
Side Scatter - Width
1.6732
0.05664
B
Data Normalization
Import processed flow data (cell count and fluorescence)
Remove samples with fewer than 1000 lymphocytes or fewer than 500 usable cells
Calculate plate median and log2 fold changes
Edge correction and recalculate log2 fold changes
1293 guides targeting 430 genes
31,209 samples over 393 plates
154 of 31,209 samples redacted
Averaging and Filtering
Calculate average and standard deviation (SD) across technical triplicates
Redact samples with low cell count (log2 fold change cell count <-1.45)
Redact highly variable samples (SD>2 log2 fold change infection or SD>1.5 log2 fold change cell count)
10,351 averages and SDs
333 of 10,351 averages redacted
Merge with sequencing data where available
83% of guides yielded mutational efficiency data
Integrateion and Hit Calling
Apply cutoff of 30% mutational efficiency
Iterative determination of log 2 fold infectivity cutoff to yield <1% false discovery
Guides must show significane in at least 2 donors OR over at least 2 timepoints
Collapse guide level data to gene level to determine list of host factors
69.7% of guides yielded >30% knock-out
log2 fold change in infection cutoffs:
Day 3 = 0.60
Day 5 = 0.56
Day 7 = 0.57
133 guides passed the threshold
86 HIV host factors
Annotate and visualize
C
Log2 fold change cell count from the median
-1.45
Redacted samples
CDK9 samples
All others
n = 1293 guides
Day 3
Day 5
Day 7
Rank Ordered Guides (x1000)

Supplementary Figure 2 | Data Analysis Strategy. a, Gating strategy for flow cytometry analysis. Representative CXCR4-targeted (right) and non-targeting control wells (left) are shown. A consistent template was used throughout analysis, with adjustment on a per-plate basis as needed. Briefly, cells were gated for lymphocytes by lightscatter followed by doublet discrimination in both side and forward scatter. Cells with equal fluorescence in the BL-1 (GFP) channel and VL-2 (AmCyan) channels were identified as auto-fluorescent and excluded from analysis. A consistent gate was then used to quantify the fraction of remaining cells that expressed GFP. **b**, Schematic of the data analysis pipeline. Briefly, individual wells were filtered for cell count and then normalized to plate median in both cell count and infection. Edge effects were corrected and plate normalization was recomputed. Technical replicates were then averaged, again filtering for low average cell count and high variability among replicates. Finally, hits were called as described. **c**, S curve plots of normalized cell count by gRNA in rank order. gRNA that were redacted from analysis due to low cell count are highlighted in red, CDK9 (a frequently redacted control) is in blue, and all others are grey. The cutoff threshold is depicted by a red dashed line.

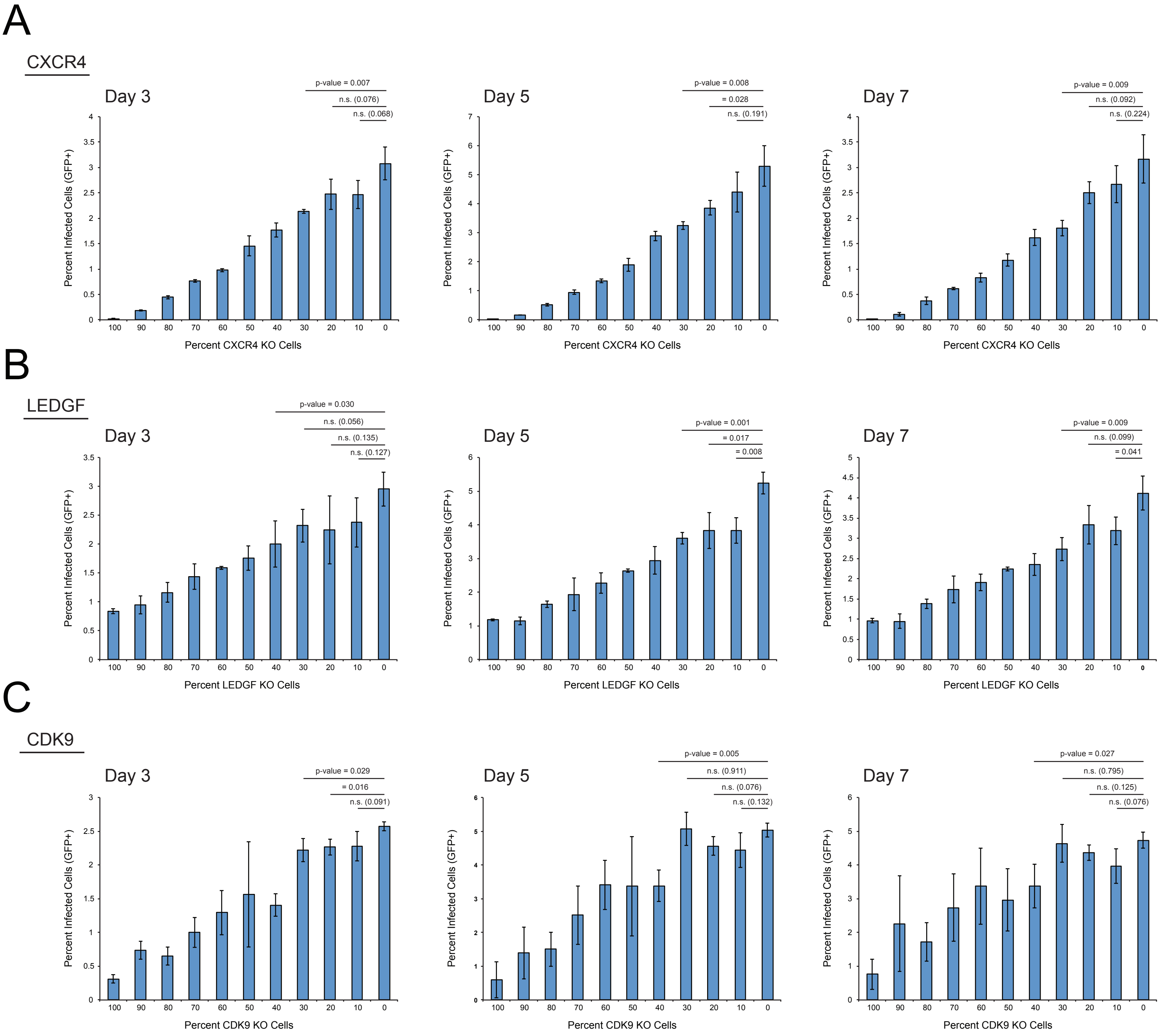
A
CXCR4
Day 3
p-value = 0.007
n.s. (0.076)
n.s. (0.068)
Percent Infected Cells (GFP+)
Percent CXCR4 KO Cells
Day 5
p-value = 0.008
= 0.028
n.s. (0.191)
Percent Infected Cells (GFP+)
Percent CXCR4 KO Cells
Day 7
p-value = 0.009
n.s. (0.092)
n.s. (0.224)
Percent Infected Cells (GFP+)
Percent CXCR4 KO Cells
B
LEDGF
Day 3
p-value = 0.030
n.s. (0.056)
n.s. (0.135)
n.s. (0.127)
Percent Infected Cells (GFP+)
Percent LEDGF KO Cells
Day 5
p-value = 0.001
= 0.017
= 0.008
Percent Infected Cells (GFP+)
Percent LEDGF KO Cells
Day 7
p-value = 0.009
n.s. (0.099)
= 0.041
Percent Infected Cells (GFP+)
Percent LEDGF KO Cells
C
CDK9
Day 3
p-value = 0.029
= 0.016
n.s. (0.091)
Percent Infected Cells (GFP+)
Percent CDK9 KO Cells
Day 5
p-value = 0.005
n.s. (0.911)
n.s. (0.076)
n.s. (0.132)
Percent Infected Cells (GFP+)
Percent CDK9 KO Cells
Day 7
p-value = 0.027
n.s. (0.795)
n.s. (0.125)
n.s. (0.076)
Percent Infected Cells (GFP+)
Percent CDK9 KO Cells

Supplementary Figure 3 | Infectivity assay sensitivity by percent edited cells and host factor. a, Time course of infection results for a population of non-targeting and CXCR4-knockout cells mixed at the indicated ratios (n = 3 technical triplicates, mean ± SD). Statistical significance was determined by two-sided pairwise Student's *t*-test. The same experiment is shown for titrations of **b**, LEDGF- and **c**, CDK9-knockout cells.

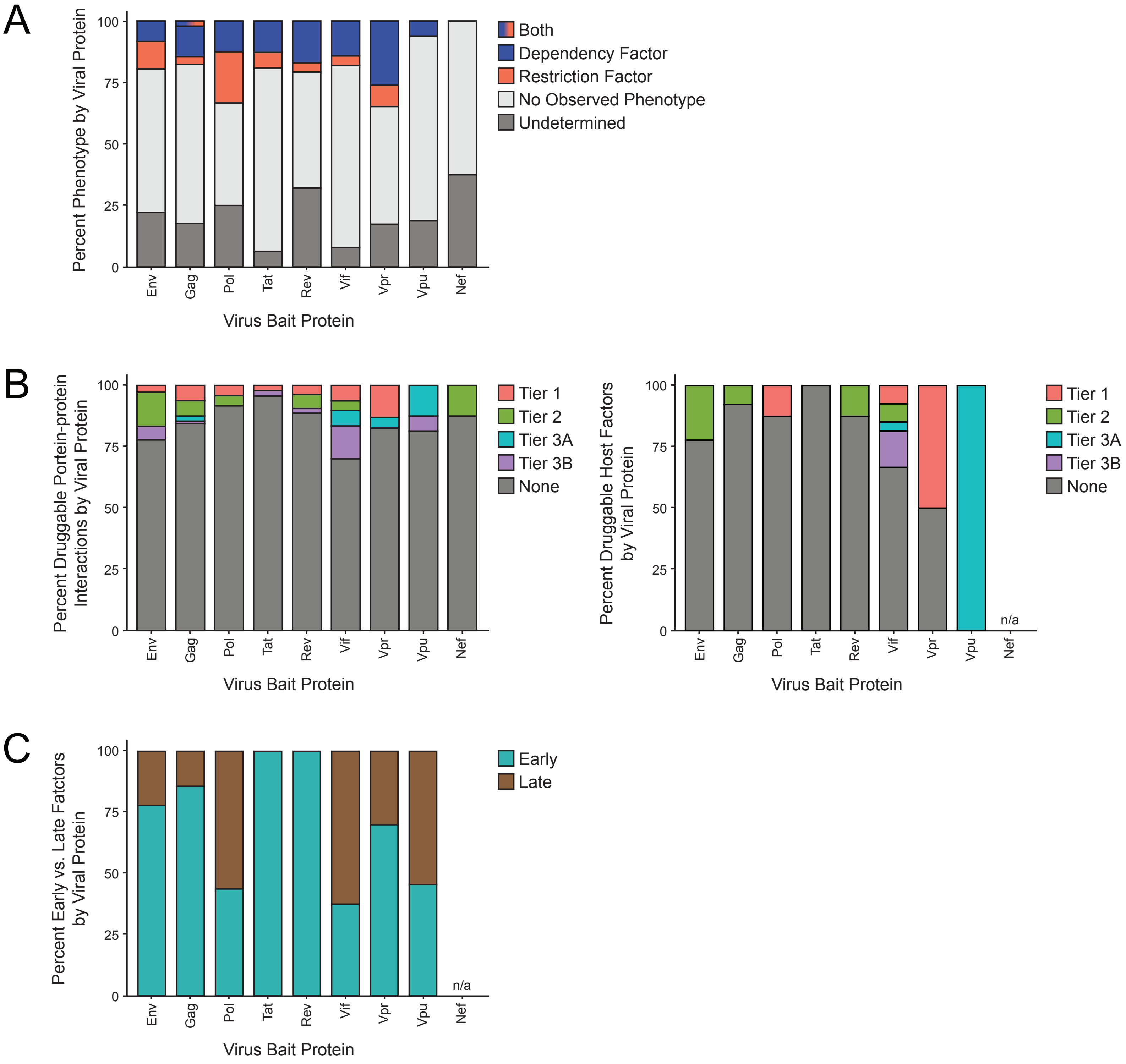
A
Percent Phenotype by Viral Protein
Both
Dependency Factor
Restriction Factor
No Observed Phenotype
Undetermined
Env
Gag
Pol
Tat
Rev
Vif
Vpr
Vpu
Nef
Virus Bait Protein
B
Percent Druggable Portein-protein Interactions by Viral Protein
Tier 1
Tier 2
Tier 3A
Tier 3B
None
Percent Druggable Host Factors by Viral Protein
n/a
C
Percent Early vs. Late Fatctors by Viral Protein
Early
Late

Supplementary Figure 4 | Classification of targeted genes by HIV polyprotein interactor and phenotype. **a**, Experimentally-determined phenotype of targeted host genes in the screen, sorted by HIV bait. **b**, Druggability of all targeted genes, according to published classification (left) and druggability of hits from the screen, according to published classification (right)[1]. **d**, Stacked cumulative bar graph of hits classified by having an early (significant on day 3) versus late (significant on days 5 or 7) phenotype by HIV bait.

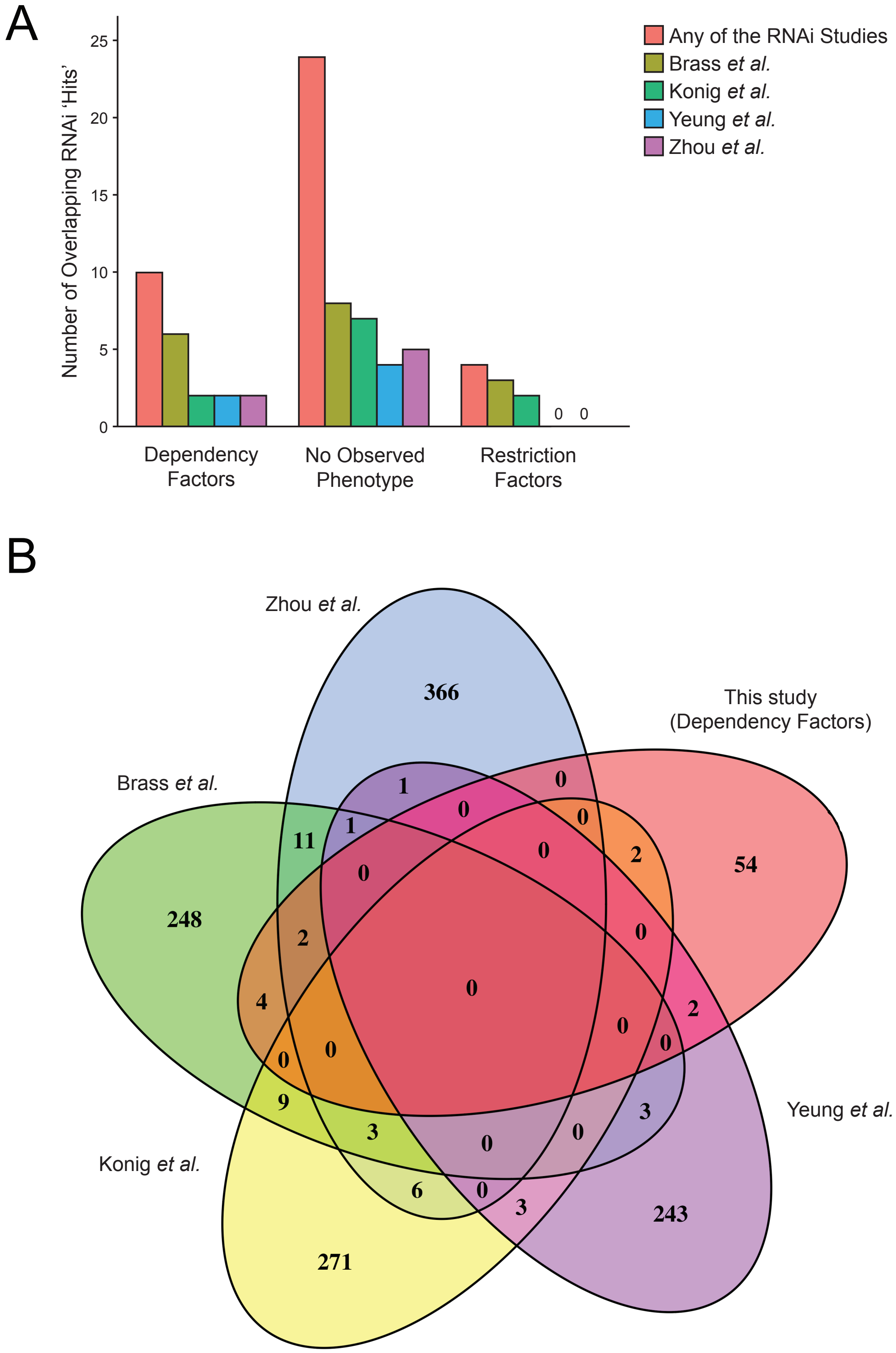
A
Number of Overlapping RNAi 'Hits'
25
20
15
10
5
0
0 0
Dependency Factors
No Observed Phenotype
Restriction Factors
Any of the RNAi Studies
Brass et al.
Konig et al.
Yeung et al.
Zhou et al.
B
Zhou et al.
This study (Dependency Factors)
Brass et al.
Konig et al.
Yeung et al.
366
54
248
271
243
1
0
0
0
1
11
0
2
0
2
0
4
0
0
2
0
0
0
9
3
3
0
0
6
0
3

Supplementary Figure 5 | Comparison of results to published genome-wide RNAi studies. **a**, Number of genes that overlap published genome-wide RNAi screens for HIV host factors, sorted by phenotype in this screen. 'Any' (red) indicates overlap with at least one of the four indicated screens. **b**, Venn diagram indicating the overlap between the candidate dependency factors identified in this screen and the results of published RNAi screens for HIV host factors. Only dependency factors were compared as none of the previously-published screens identified significant restriction factors, and only one reported perturbations that led to increased HIV replication[2].

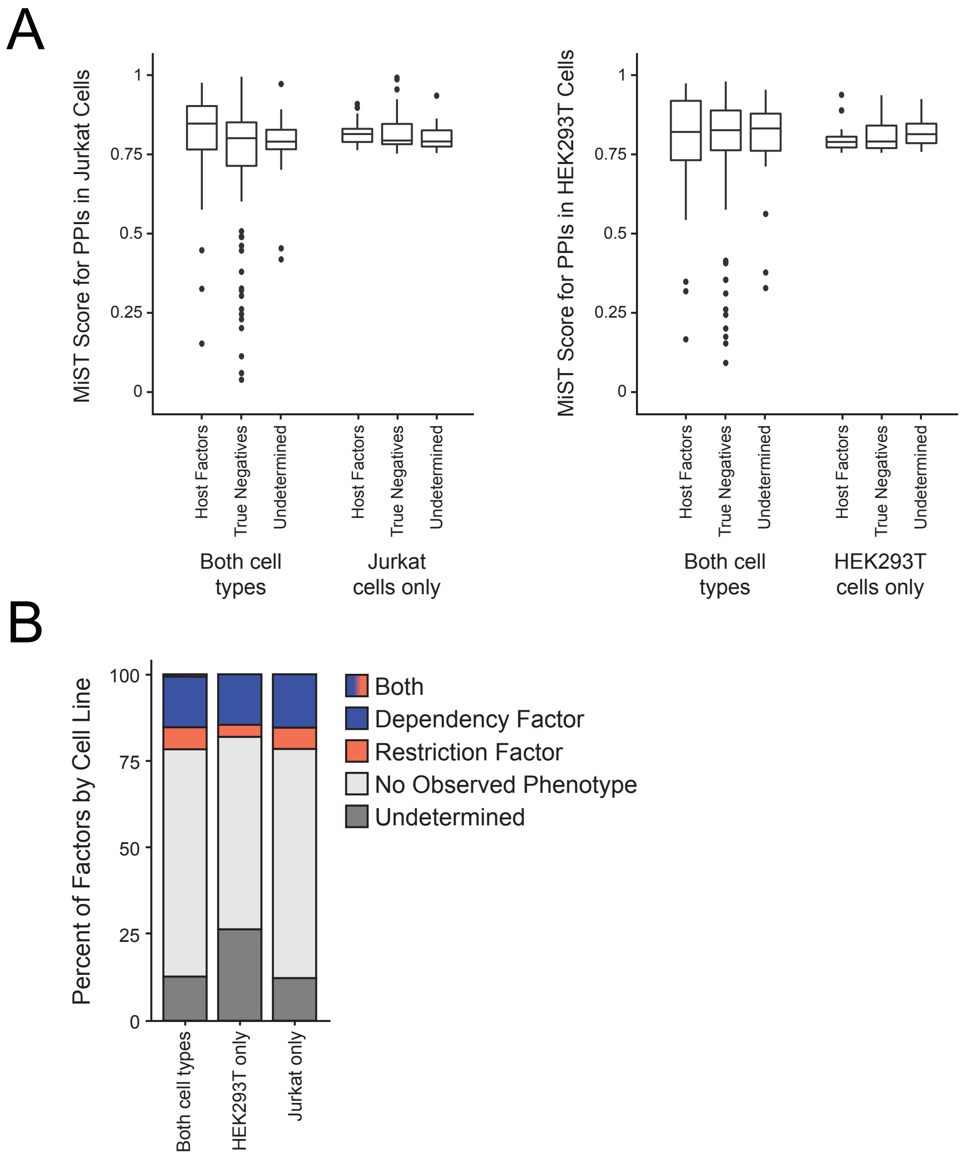
A
MiST Score for PPIs in Jurkat Cells
1
0.75
0.5
0.25
0
Host Factors
True Negatives
Undetermined
Host Factors
True Negatives
Undetermined
Both cell types
Jurkat cells only
MiST Score for PPIs in HEK293T Cells
1
0.75
0.5
0.25
0
Host Factors
True Negatives
Undetermined
Host Factors
True Negatives
Undetermined
Both cell types
HEK293T cells only
B
Percent of Factors by Cell Line
100
75
50
25
0
Both cell types
HEK293T only
Jurkat only
Both
Dependency Factor
Restriction Factor
No Observed Phenotype
Undetermined

Supplementary Figure 6 | Analysis of proteomic parameters by genetic phenotype. a, The distribution of reported proteomic probability scores calculated by the MiST algorithm categorized by HIV phenotype and cell type[3]. Center line, median; box limits, upper and lower quartiles; whiskers, 1.5x interquartile range; black points, outliers. **b**, Cumulative distribution of HIV phenotypes by the cell line in which the protein interaction was identified.

SUPPLEMENTARY REFERENCES

1 Finan, C. *et al.* The druggable genome and support for target identification and validation in drug development. *Sci Transl Med* **9**, doi:10.1126/scitranslmed.aag1166 (2017).

2 Zhou, H. *et al.* Genome-scale RNAi screen for host factors required for HIV replication. *Cell Host Microbe* **4**, 495-504, doi:10.1016/j.chom.2008.10.004 (2008).

3 Jager, S. *et al.* Global landscape of HIV-human protein complexes. *Nature* **481**, 365-370, doi:10.1038/nature10719 (2011).
