# Supplementary material for: A functional map of HIV-host interactions in primary human T cells
Source: Nat Commun. 2022 Apr 1;13:1752. doi: 10.1038/s41467-022-29346-w (PMC8976027; doi:10.1038/s41467-022-29346-w)
Supplement: Supplementary file 2 — Description of Supplementary Files [file 41467_2022_29346_MOESM2_ESM.docx]

**DESCRIPTION OF ADDITIONAL SUPPLEMENTARY FILES**

File Name: **Supplementary Data 1**

Description: **Sequences, average mutational efficiency, and predicted off-target scores for all gRNA used in this study.** *Count*: Count of guide RNA (gRNA) used in the initial screen. *Dharmacon Catalog Number*: Reference number for Edit-R knockout guide, or ‘control’ for custom-designed CXCR4, LEDGF and CDK9 targeting gRNA; *Gene Name*: Gene symbol of targeted gene, or ‘NTC’ for non-targeting control; *Gene ID*: Entrez Gene ID of targeted gene, or ‘NA’ for non-targeting negative controls; *Guide Sequence*: 20 base pair protospacer sequence of each gRNA; *Guide ID*: Unique name for each gRNA used in this study; *Average mutational efficiency*: Average mutational efficiency across all donors where sequencing passed quality filtering, or ‘NA’ if no sequencing data was available; *Top5* and *Top10 Offtarget score*: Total computed OutScore^4^ of the top 5 and top 10, respectively, predicted off-target sites for the guide sequence as generated by CRISPRseek^5^. Specific guides could not be synthesized for nine genes previously reported in^3^: *EIF3C*, *CKMT1B*, *YWHAZ*, *SMN2*, *ICAM2*, *HSPA7*, *HSPA6*, *FAM133B*, and *PSMD11*.

File Name: **Supplementary Data 2**

Description: **List of individual points that reached the significance threshold.** *Donor*: Unique ID (alphanumeric) of the healthy human donor that was used for indicated knockout; *Day*: Days post-infection after which the sample was taken (3, 5, or 7); *Guide ID*: Unique identifier for the gRNA, matches the field in **Supplementary Data 1**; *Well*: Location in the 96-well array, numeric component is always two digits; *Average log_2_ fold change in infection*: Mean of technical triplicate log_2_ fold change in infection relative to plate median, negative infinite values were imputed to -12; *SD of log_2_ fold change in infection*: Standard deviation of technical triplicate log_2_ fold change in infection relative to plate median; *Average log_2_ fold change in cell count*: Mean of technical triplicate log_2_ fold change in cell count relative to plate median; *SD of log_2_ fold change in cell count*: Standard deviation of technical triplicate log_2_ fold change in cell count relative to plate median; *Factor Type*: D for dependency factors (decrease in infection relative to median) and R for restriction factors (increase in infection relative to median).

File Name: **Supplementary Data 3**

Description: **Initial screen results by gene with annotation.** *Count*: Count of genes in the initial study based on the HIV-human PPIs identified in^3^; *Gene ID*: Gene symbol; *HIV Bait*: HIV protein or polyprotein with which each host factor was found to be associated^3^ (Note: polyproteins are preferentially listed, *i.e.* NC-interactors are listed as Gag; *Observed Phenotype*: Final call for all genes tested in this study as one of the following: ‘TRUENEG’ - successful knockout with no observed HIV phenotype, ‘D’ - Dependency factor, ‘R’ - Restriction factor, ‘UNDETERMINED’ - insufficient level of knockout for phenotypic assessment; *Early phenotype*: ‘NA’ - target is neither a dependency or a restriction factor, ‘TRUE’ - target is a host factor that passed the infectivity threshold at day 3, ‘FALSE’ - target is a host factor that did not pass the infectivity threshold until after day 3; *Druggability*: Druggability tier or ‘NA’ if the target is not represented in the druggability data set^1^; *Known*: ‘TRUE’ - functional role for the target gene in HIV infection has been previously described in the literature, ‘FALSE’ - functional role for the target gene in HIV infection has not been previously described in the literature (See also **Supplementary Data 5**); *Number of Donors*: Number of human blood donors in which this gene was targeted for editing; *Cell Line*: Cell type in which the gene target was originally identified as an HIV-human PPI^3^: ‘HEK293T’, ‘Jurkat’, ‘Both’, or ‘NA’ for non-PPI controls; *HEKScore*: Previously determined MiST score of the HIV-human PPI in HEK293T cells from 0 to 1, higher scores indicate more confidence^3^; JurkatScore: Previously determined MiST score of the HIV-human PPI in Jurkat cells from 0 to 1, higher scores indicate more confidence^3^; *Donor Dependent?*: ‘NA’ - target is not a hit, ‘TRUE’ - targeted gene yields a phenotype in less than 50% of tested donors, ‘FALSE’ - targeted gene yields a phenotype in greater than 50% of tested donors.

File Name: **Supplementary Data 4**

Description: **Literature review of genes targeted in this study.** *GENEID*: Entrez Gene ID for target gene; *UNIPROT*: Uniprot identifier for protein encoded by target gene; *Gene Symbol*: Gene symbol of target gene; *Functional Role in HIV Biology?*: ‘YES’ - established functional role for target gene in HIV replication, excluding previous genome-wide screens, ‘NO’ - no functional role previously ascribed to target gene, excluding previous genome-wide screens; *Relevant PubMed IDs*: Non-exhaustive list of PMIDs of papers describing the role of the target gene in HIV replication, most pulled from GeneRIFs; *Notes*: Non-exhaustive list of described roles extracted from GeneRIFs and manually curated from PubMed. Following sheet describes the literature review process used.

File Name: **Supplementary Data 5**

Description: **Phylogenetic Analysis by Maximal Likelihood (PAML) results sorted in order of statistical significance.** *Gene name:* Gene symbol of target gene; *Positive selection previously known:* For genes showing evidence of positive selection, ‘Yes’ - gene reported to be under positive selection in the literature, ‘No’ - gene not reported to be under positive selection in the literature; *Category*: Phenotypic call in the initial screen as a dependency factor, restriction factor, or both; *Number of aligned sequences:* Number of gene orthologs aligned and included in the analysis; *Alignment length (codons):* Number of codons aligned for evolutionary analysis in each gene; *Model 8 vs. 8a uncorrected p-value:* Uncorrected p-value (likelihood ratio test, chi-squared distribution with 1 degree of freedom) for the likelihood of a given gene to be under positive selection after Phylogenetic Analysis by Maximal Likelihood (PAML) comparing an evolutionary model that allows for a subset of codons evolving under positive selection (‘Model 8’) versus a model that allows only neutral and purifying selection (‘Model 8a’); *Model 8 vs. 8a BH-adjusted p-value:* Adjusted p-value following the Benjamini-Hochberg procedure to control the false discovery rate; *Percent of sites under positive selection:* Percent of codons in the indicated gene with evidence of positive selection; *dN/dS of positively-selected sites:* Ratio of nonsynonymous to synonymous substitutions observed across sites under positive selection; *Number of codons with high likelihood of being in positively-selected class (BEB>=0.9):* Number of codons in the gene likely to be under positive selection following Bayes empirical bayes (BEB) estimation*; List of sites likely to be under positive selection (BEB>=0.9):* Amino acid residues most likely to be under positive selection (position numbering refers to alignment); *CpG-masked alignment length (codons):* Number of codons aligned for evolutionary analysis in each gene following CpG masking*; CpG-masked alignment, Model 8 vs. 8a uncorrected p-value:* Uncorrected p-value (likelihood ratio test, chi-squared distribution with 1 degree of freedom) for the likelihood of a given gene to be under positive selection after PAML comparing Model 8 and Model 8a*;CpG-masked alignment, Model 8 vs. 8a BH-adjusted p-value:* Adjusted p-value following the Benjamini-Hochberg procedure to control the false discovery rate.

File Name: **Supplementary Data 6**

Description: **Raw infection data.** *Count*: Running count of flow cytometry results in the analysis; *Well*: Location in the 96-well array, numeric component is always two digits; *pcLymphos*: Percent of cells in the lymphocyte gate by lightscatter; *ctLymphos*: Number of cells in the lymphocyte gate by lightscatter; *pcGFP*: Percent of non-autofluorescent lymphocytes that are GFP+; *ctGFP*: Number of non-autofluorescent lymphocytes that are GFP+; *pcAutofluor*: Percent of lymphocytes that are autofluorescent; *ctAutofluor*: Number of lymphocytes that are autofluorescent; plate: Unique 96-well plate identifier; *cr*: Plate map ID, all plates with the same cr have the same guides in the same wells; *dnr*: Unique donor ID; *day*: Timepoint (3, 5, or 7 days post-infection); *targetgene*: Gene symbol of the target; *guide*: Unique identifier for the gRNA, matches the ‘Guide ID’ field in **Supplementary Data 1**; *ctGoodCells*: Number of cells in the lymphocyte gate that are not autofluorescent.

File Name: **Supplementary Data 7**

Description: **Averaged infection data.** *Count*: Running count of individual perturbations in the analysis; *cr*: Plate map ID, all plates with the same cr have the same guides in the same wells; *dnr*: Unique donor ID; *day*: Timepoint (3, 5, or 7 days post-infection); *guide*: Unique identifier for the gRNA, matches the same field in **Supplementary Data 6** and the ‘Guide ID’ field in **Supplementary Data 1**; *well*: Location in the 96-well array, numeric component is always two digits; *targetgene*: Gene symbol of the target gene; *bait*: HIV protein with which the given host factor was found to associate^3^; *avg.logfoldmed*: Average log_2_ fold change in infection relative to the plate median of technical triplicates; *sd.logfoldmed*: Standard deviation of the log_2_ fold change in infection relative to the plate median of technical triplicates; *avg.foldmed*: Average fold change in infection relative to the plate median of technical triplicates; *sd.foldmed*: Standard deviation of the fold change in infection relative to plate median of technical triplicates; *avg.logfoldctplate*: Log_2_ fold change in live cell count relative to plate median, average of technical triplicates; *sd.logfoldctplate*: Standard deviation of technical triplicates of log_2_ fold change in cell count relative to plate median.

File Name: **Supplementary Data 8**

Description: **Averaged infection data for multiplex validation.** *Gene*: Gene symbol of the target gene; *Day*: Timepoint (3, 5, or 7 days post-infection); *Avg_Log2FC*: Average log_2_ fold change in infection relative to plate median across three biological replicates (cells from three independent human blood donors); *Dnr1_Log2FC*: Average log_2_ fold change in infection relative to plate median across three technical replicates within a single human blood donor (Donor 1); *Dnr2_Log2FC*: Average log_2_ fold change in infection relative to plate median across three technical replicates within a single human blood donor (Donor 2); *Dnr3_Log2FC*: Average log_2_ fold change in infection relative to plate median across three technical replicates within a single human blood donor (Donor 3); *SE*: Standard error of the average log_2_ fold change in infection relative to plate median across the three biological replicates; *expected*: The phenotype predicted for the gene from the initial screen results, ‘D’ - dependency factor, ‘R’ - restriction factor, ‘toxic’ – essential gene following viability staining; *pvalue_WRS*: p-value after two-sided Wilcoxon Rank Sum test for significance; *adj.pvalue_WRS*: Adjusted p-value after two-sided Wilcoxon Rank Sum test for significance (Benjamini-Hochberg procedure^6^ to control the false-discovery rate).

**SUPPLEMENTARY REFERENCES**

1 Finan, C. *et al.* The druggable genome and support for target identification and validation in drug development. *Sci Transl Med* **9**, doi:10.1126/scitranslmed.aag1166 (2017).

2 Zhou, H. *et al.* Genome-scale RNAi screen for host factors required for HIV replication. *Cell Host Microbe* **4**, 495-504, doi:10.1016/j.chom.2008.10.004 (2008).

3 Jager, S. *et al.* Global landscape of HIV-human protein complexes. *Nature* **481**, 365-370, doi:10.1038/nature10719 (2011).

4 Hsu, P. D. *et al.* DNA targeting specificity of RNA-guided Cas9 nucleases. *Nat Biotechnol* **31**, 827-832, doi:10.1038/nbt.2647 (2013).

5 Zhu, L. J., Holmes, B. R., Aronin, N. & Brodsky, M. H. CRISPRseek: a bioconductor package to identify target-specific guide RNAs for CRISPR-Cas9 genome-editing systems. *PLoS One* **9**, e108424, doi:10.1371/journal.pone.0108424 (2014).

6 Benjamini, Y. & Yekutieli, D. Quantitative trait Loci analysis using the false discovery rate. *Genetics* **171**, 783-790, doi:10.1534/genetics.104.036699 (2005).
