# Supplementary material for: A functional map of HIV-host interactions in primary human T cells
Source: Nat Commun. 2022 Apr 1;13:1752. doi: 10.1038/s41467-022-29346-w (PMC8976027; doi:10.1038/s41467-022-29346-w)
Supplement: Supplementary file 11 — Reporting Summary [file 41467_2022_29346_MOESM11_ESM.pdf]

nature portfolio

Corresponding author(s): Nevan J. Krogan
Alexander Marson
Judd F. Hultquist

Last updated by author(s): January 26, 2022

Reporting Summary

Nature Portfolio wishes to improve the reproducibility of the work that we publish. This form provides structure for consistency and transparency in reporting. For further information on Nature Portfolio policies, see our Editorial Policies and the Editorial Policy Checklist.

Statistics

For all statistical analyses, confirm that the following items are present in the figure legend, table legend, main text, or Methods section.

n/a	Confirmed	
☐	☒	The exact sample size (*n*) for each experimental group/condition, given as a discrete number and unit of measurement
☐	☒	A statement on whether measurements were taken from distinct samples or whether the same sample was measured repeatedly
☒	☐	The statistical test(s) used AND whether they are one- or two-sided<br>*Only common tests should be described solely by name; describe more complex techniques in the Methods section.*
☒	☐	A description of all covariates tested
☐	☒	A description of any assumptions or corrections, such as tests of normality and adjustment for multiple comparisons
☐	☒	A full description of the statistical parameters including central tendency (e.g. means) or other basic estimates (e.g. regression coefficient) AND variation (e.g. standard deviation) or associated estimates of uncertainty (e.g. confidence intervals)
☐	☒	For null hypothesis testing, the test statistic (e.g. *F*, *t*, *r*) with confidence intervals, effect sizes, degrees of freedom and *P* value noted<br>*Give P values as exact values whenever suitable.*
☒	☐	For Bayesian analysis, information on the choice of priors and Markov chain Monte Carlo settings
☒	☐	For hierarchical and complex designs, identification of the appropriate level for tests and full reporting of outcomes
☒	☐	Estimates of effect sizes (e.g. Cohen's *d*, Pearson's *r*), indicating how they were calculated

Our web collection on statistics for biologists contains articles on many of the points above.

Software and code

Policy information about availability of computer code

Data collection	Primer design: Primer3 (github.com/czbiohub/Primer3Wrapper)<br>Flow Cytometry: Attune NxT Software v3.2.0
Data analysis	Software used for the analysis of these data are as follows:<br>FlowJo v10.5.3 (TreeStar)<br>Bioconductor (Release 3.14) - CrispRVariants<br>RStudio v1.4<br>MACSE v2.00<br>phyml v3.0<br>PAML v4.9<br><br>Custom code for the analysis of editing efficiency is available through SRA (BioProject: PRJNA486372) and Figshare (https://doi.org/10.6084/m9.figshare.6957119.v1). Likewise, custom code used for statistical analysis has been uploaded to Figshare (https://doi.org/10.6084/m9.figshare.7246652).

For manuscripts utilizing custom algorithms or software that are central to the research but not yet described in published literature, software must be made available to editors and reviewers. We strongly encourage code deposition in a community repository (e.g. GitHub). See the Nature Portfolio guidelines for submitting code & software for further information.

Data

Policy information about availability of data

All manuscripts must include a data availability statement. This statement should provide the following information, where applicable:

- Accession codes, unique identifiers, or web links for publicly available datasets
- A description of any restrictions on data availability
- For clinical datasets or third party data, please ensure that the statement adheres to our policy

DATA AVAILABILITY STATEMENT
All raw sequencing data and downstream analyses are openly available through SRA (BioProject: PRJNA486372) and Figshare (https://doi.org/10.6084/m9.figshare.6957119.v1). All raw and processed flow cytometry data, mutational efficiency data, and gRNA sequences are provided here as Supplemental Tables. All other data are available from the corresponding author upon reasonable request.

Field-specific reporting

Please select the one below that is the best fit for your research. If you are not sure, read the appropriate sections before making your selection.

[x] Life sciences [ ] Behavioural & social sciences [ ] Ecological, evolutionary & environmental sciences

For a reference copy of the document with all sections, see nature.com/documents/nr-reporting-summary-flat.pdf

Life sciences study design

All studies must disclose on these points even when the disclosure is negative.

Sample size	All knock-out experiments were performed in T cells isolated from 2-7 independent human blood donors as reported in the supplemental tables and figure legends. The rationale was to complete all experiments in at least two independent donors with additional donors being run dependent on cell availability. Knock-outs were performed with three independent guide RNA per gene and validated by amplicon sequencing. The use of 3-5 independent guide RNA is typical for most CRISPR screens in the field, and our data validation data reported here justify the high efficacy of at least one of three guides. All infections were performed in technical triplicate. Again, this is standard in the field to capture technical variability in the infection protocol.
Data exclusions	HIV infection data reported in Figure 1 were excluded if cell count was too low in a given well due to biological function (i.e., a gene targeted for knock-out was essential and the cells died) or due to technical error (i.e., an instrument clog). Exclusion criteria are noted in the text.
Replication	Again, all knock-out experiments were performed in 2-7 biological replicates with 3-5 independent guide RNA. All infection experiments were conducted in technical triplicate. Variance across all controls is reported in Figure 1. Variance in guide efficacy is reported in Figure 2 with all raw data provided in the Supplement. Biological variability observed between donors is reported alongside magnitude in Figure 3. All 86 candidates were subject to validation in 3 independent donors using a distinct, multiplexed pool of 4 guide RNA as reported in Figure 6. Of these, 13 showed evidence of toxicity, 47 confirmed, and 26 failed to confirm using appropriate statistical tests as discussed extensively in the text.
Randomization	Guide RNA targeting a single gene were always on the same single 96-well plate and cells isolated from a single donor were used for each plate. This allowed for the calculation of all infectivity data relative to the plate median to account for donor and plate variability. Genes were arrayed randomly onto the 96-well plates alongside the same 6 standard controls as reported in Figure 1. Donors were randomly assigned to each plate of guides depending on the number of cells available with at least two independent donors being assigned for each plate. All other experiments and analyses beyond this initial screen were conducted as one group and randomization was not necessary.
Blinding	All cells were provided from anonymous donors following de-identification. All plates of guide RNA were likewise assigned plate numbers that gave no indication of gene identity in each well. Control guides were assigned to the same wells on all plates so that technical staff could perform technical validation in real time. Otherwise, the identity of each gene being targeted in each experiment was masked until integration of plate layouts upon analysis.

Reporting for specific materials, systems and methods

We require information from authors about some types of materials, experimental systems and methods used in many studies. Here, indicate whether each material, system or method listed is relevant to your study. If you are not sure if a list item applies to your research, read the appropriate section before selecting a response.

Materials & experimental systems

n/a	Involved in the study	
☐	☒	Antibodies
☐	☒	Eukaryotic cell lines
☒	☐	Palaeontology and archaeology
☒	☐	Animals and other organisms
☐	☒	Human research participants
☒	☐	Clinical data
☒	☐	Dual use research of concern

Methods

n/a	Involved in the study	
☒	☐	ChIP-seq
☐	☒	Flow cytometry
☒	☐	MRI-based neuroimaging

Antibodies

Antibodies used

LEDGF (1:2000 dilution, clone C57G11, Cell Signaling Technologies, Cat. No. 2088S), CCNT1 (1:1000 dilution, clone D1B6G, Cell Signaling Technologies, Cat. No. 81464S), and CYPA (1:12000 dilution, polyclonal, Cell Signaling Technologies, Cat. No. 2175S) levels were probed relative to b-actin (1:10000 dilution, clone 8H10D10, Cell Signaling Technologies, Cat. No. 3700S) as a protein loading control. Anti-rabbit or anti-mouse IgG horseradish peroxidase (HRP)-conjugated secondary antibodies (1:20000, polyclonal, Jackson ImmunoResearch Laboratories, Cat. Nos. 111-035-003 and 115-035-003) were detected using Pierce™ ECL Western Blotting Substrate (ThermoFisher).

Validation

All antibodies provided from authenticated, commercial sources as listed above. All antibodies have been approved for use in Western blotting approaches by the manufacturer at the dilutions specified (https://www.cellsignal.com/).

Eukaryotic cell lines

Policy information about cell lines

Cell line source(s)

HEK293T cells (ATCC, CRL-3216)
HeLa-TZM (AIDS Reagent Program #1470)

Authentication

Cells were received direct from the supplier with certificate of validation. No additional cell line authentication was performed in house.

Mycoplasma contamination

All cell line stocks were checked for mycoplasma twice per year by PCR. No mycoplasma was detected.

Commonly misidentified lines (See ICLAC register)

No commonly misidentified cell lines were used in this study.

Human research participants

Policy information about studies involving human research participants

Population characteristics

The healthy volunteers consist of individuals age 18-60 years old, weighing at least 110 pounds (per self report). All participants are required to complete an informed consent or assent form. Although not a formal exclusion, participants may be asked if they have a history of immune-mediated disease or infection or are taking immune suppressant drugs at the time of the study, or if women are pregnant or lactating. These factors would not necessarily exclude participants, but would affect interpretation of some functional studies performed for this protocol. Samples are de-identified after draw and were randomized for assignment to plates. Cells from equal numbers of men and women were included in this study. Cells were additionally sourced from StemCell, which were provided to the team in a de-identified fashion. Inclusion criteria for those patients were determined by the provider (https://www.stemcell.com/products/product-types/primary-and-cultured-cells/lrs-cone.html).

Recruitment

Potential participants were recruited via advertisements, notices, and/or other forms of media. Interested subjects initiated contact with study investigators. In addition, volunteers known to study team (directly or indirectly) were recruited through word of mouth, email, text message, social media and/or phone.

Ethics oversight

UCSF Committee on Human Research (CHR #13-11950)

Note that full information on the approval of the study protocol must also be provided in the manuscript.

Flow Cytometry

Plots

Confirm that:

- [x] The axis labels state the marker and fluorochrome used (e.g. CD4-FITC).
- [x] The axis scales are clearly visible. Include numbers along axes only for bottom left plot of group (a 'group' is an analysis of identical markers).
- [x] All plots are contour plots with outliers or pseudocolor plots.
- [x] A numerical value for number of cells or percentage (with statistics) is provided.

Methodology

Sample preparation	At days 3, 5, and 7 post-infection, 75 µL of each cell suspension was removed and mixed 1:1 with freshly made 2% formaldehyde in PBS (Sigma) and stored at 4°C. Samples were run on the flow cytometer at least 30 minutes (and no more than 2 weeks) after fixation.
Instrument	ThermoFisher Attune NxT
Software	FlowJo 10.5.3
Cell population abundance	Described in full in Supplemental Figure 2. Briefly, viable cells made up roughly 75% of all events monitored, 99% of which were singlets. Cells generally ranged from 0 to 5% GFP+ depending on the donor, perturbation, and days post-infection.
Gating strategy	Described in full in Supplemental Figure 2. Briefly, a live cell gate based on FSC/SSC axes was used followed by two successive singlet gates (SSC - Height versus SSC - Width, FSC-Height versus FSC-Width). Autofluroescent cells were then excluded by gating on cells with equivalent excitation in the violet and blue (GFP) channels. GFP+ cells were defined as cells in this final population with GFP signal above 700.

- [x] Tick this box to confirm that a figure exemplifying the gating strategy is provided in the Supplementary Information.
